# Coulomb stress analysis for several filling and operational scenarios at the Grand Ethiopian Renaissance Dam impoundment

**DOI:** 10.1007/s12665-021-09591-w

**Published:** 2021-03-28

**Authors:** Austin Madson, Yongwei Sheng

**Affiliations:** Department of Geography, University of California, Los Angeles (UCLA), 1255 Bunche Hall, Box 951524, Los Angeles, CA 90095 USA

**Keywords:** Hydrologic loading, Coulomb stress, Grand Ethiopian Renaissance Dam, Reservoir triggered seismicity, Cluster computing

## Abstract

Increased demand for power generation coupled with changing seasonal water uncertainty has caused a worldwide increase in the construction of large hydrologic engineering structures. That said, the soon-to-be-completed Grand Ethiopian Renaissance Dam (GERD) will impound the Blue Nile River in Western Ethiopia and its reservoir will encompass ~ 1763 km^2^ and store ~ 67 Gt (km^3^) of surface water. The impoundment will undergo maximum seasonal load changes of ~ 28 to ~ 36 Gt during projected seasonal hydroelectric operations. The GERD impoundment will cause significant subsurficial stresses, and could possibly trigger seismicity in the region. This study examines Coulomb stress and hydrologic load centroid movements for several GERD impoundment and operational scenarios. The maximum subsurficial Coulomb stress applied on optimally oriented fault planes from the full impoundment is ~ 186 kPa and over 30% of our model domain incurs Coulomb stresses ≥ 10 kPa, regardless of the impoundment period length. The main driver behind Coulomb stress and load centroid motion during impoundment is the annual, accumulated daily reservoir storage change. The maximum Coulomb stresses from the highest amplitude season of five long-term operational scenarios are around 36, 33, 29, 41, and 24% of the total maximum stresses from the entire GERD impoundment. Variations in annual Coulomb stresses during modeled GERD operations are attributed to the seasonal load per unit area, and partially to the initial seasonal water level. The spatial patterns and amplitudes of these stress tensors are closely linked to both the size and timing of GERD inflow/outflow rates, and an improved understanding of the magnitude and extent of these stresses provides useful information to water managers to better understand potential reservoir triggered seismic events from several different operational and impoundment strategies.

**Supplementary information:**

The online version contains supplementary material available at 10.1007/s12665-021-09591-w.

## Introduction

Large scale hydrologic loading and reservoir operations have the ability to affect the surrounding lithosphere and crust as well as the interconnected hydrogeological systems. To this end, large impoundments and reservoir operational cycles would alter local potentiometric groundwater surface elevations. That said, impoundments could cause seepage to adjacent subsurface rocks as well as into hydraulically connected aquifers. This surface water diffusion into adjacent subsurficial rocks is able to increase pore pressure, which in turn can reduce the frictional stress. To that end, past research has shown that the initial impoundment and subsequent reservoir operations of large dams have altered groundwater levels for hydraulically connected systems (Zhang et al. 2014; Zhao et al. 2016). These connections have the capability to destabilize slopes and to eventually trigger failure events (Teimouri and Khalkhali 2018). This instability is mostly caused by variations in hydrostatic pressure from the changing groundwater levels and the fluctuations in the hydraulically connected water levels of the reservoir (Fredlund and Rahardjo 1993; Paronuzzi et al. 2013; Xia et al. 2015; Zhang et al. 2012).

Additional impacts from large impoundments are associated with the large hydrologic storage fluxes that apply notable force on to the Earth’s surface. Extreme changes in reservoir storage caused by large hydrological engineering construction projects (e.g. the Aswan High and the Three Gorges Dam) are likely to cause increases in both strain and stress on nearby fault systems and can lead to an increase in seismic events (Allen 1982; Chander and Chander 1996; Gahalaut et al. 2018; Ge et al. 2009; Kerr and Stone 2009; Talwani 1997). Initial impoundment as well as seasonal fluctuations in reservoir water levels during operational phases from these hydro-engineering projects will cause notable fluctuations in surface water extent and reservoir volume. These vast changes in water loads can create non-negligible forces on the Earth's surface and are also capable of deforming or displacing the adjacent lithosphere (Madson and Sheng 2020). To that end, many researchers have proven that both remotely sensed and in situ data products (e.g. GRACE, GNSS, InSAR, etc.) have the capability to quantify the flexural response from changes in hydrologic loads (drought, lakes, regional climatic changes, reservoirs, seasonal precipitation, snow, etc.) (Borsa et al. 2014; Dumka et al. 2018; Enzminger et al. 2018; Gahalaut et al. 2017; Kraner et al. 2018; Madson et al. 2017; Neelmeijer et al. 2018; Tregoning et al. 2009). The vast size of large hydro-engineering projects creates a scenario where marked changes in surface water loads can occur during both the initial impoundment phases and the annual hydrologic operational periods. The size and timing of these water storage changes are mostly determined by the initial impoundment and annual operational policies that the GERD's water managers select. These initial impoundment scenarios along with the annual hydrological operation scenarios play vastly important roles with respect to the application of surface water load induced lithospheric stresses for hydrologic engineering projects with large impoundment volumes.

A drastic influx of water into a reservoir can apply large stresses on the region as well as significantly increase the pore pressure in the surrounding areas (Simpson 1976). Dozens of cases of post-impounding seismicity have been researched over the last several decades, and these topics are of great concern for large reservoirs (Baisch et al. 2006; Bell and Nur 1978; Chen and Talwani 1998; Gahalaut et al. 2007; Ghaboussi and Wilson 1973; Gupta 2002; Gupta et al. 2000; Mekkawi et al. 2004; Roeloffs 1988; Simpson and Negmatullaev 1981; Talwani and Acree 1985; Tao et al. 2015; Zoback and Hickman 1982). However, understanding these reservoir triggered seismic (RTS) events is not straightforward. For example, in some cases the increased RTS activity occurs during the filling stages, while other large reservoir projects have documented increases only after an impoundment is complete and several seasonal operational phase cycles have been completed (Simpson et al. 1988; Talwani 1997). There are two dominant mechanisms responsible for RTS: (1) increased normal and shear stress from the elastic response to the hydrologic loading and/or unloading and (2) increased pore pressure from a reduction in effective normal stresses (Bell and Nur 1978; Roeloffs 1988; Simpson 1986; Simpson et al. 1988; Snow 1972; Talwani 1997). However, the stress changes from the elastic response can also be a stabilizing factor for the underlying and reservoir-adjacent regions, but this is dependent on the overall geometry of the impoundment relative to nearby faults as well as the preexisting stressors in the study area (Rajendran and Talwani 1992; Tao et al. 2015).

This study will focus on changes in normal (*σβ*) and shear (*τβ*) stresses brought on by different impoundment scenarios and seasonal operations at the notable Grand Ethiopian Renaissance Dam (GERD) impoundment site. More specifically, this work examines changes in Coulomb stress from the GERD’s hydrologic load on optimally oriented planes in an elastic half-space. Typically, fault plane failures occur when Coulomb stresses exceed a certain threshold (Harris 1998; King et al. 1994; Stein 1999). That said, a robust analysis of spatiotemporal changes in Coulomb stress helps to provide a meaningful assessment on the potential of triggered seismic events from different reservoir impoundment and operational strategies. These changes in the Coulomb stress state are mostly dependent on the frequency and amplitude of the reservoir fluxes as they relate to the initial filling stages as well as the subsequent operation of the reservoir. This highlights the need for a better understanding of the predicted subsurficial stress fields at the GERD as it relates to the creation of a well thought out and appropriately timed impoundment/filling strategy along with a reasonable operational reservoir cycle.

The goal of this research is to provide an initial analysis of the Coulomb stresses applied on optimal fault planes as imposed by many different impoundment strategies and seasonal reservoir release policies at the GERD. We undertook this analysis to increase the understanding of hydrologic load induced stress at the site of the GERD impoundment. To that end, the specific goals of this work are to provide meaningful answers to the following science questions: (1) What are the Coulomb stresses at depth on optimally oriented fault planes as caused by hydrologic load changes from different filling schedules as well as from several seasonal operation plans at the GERD? (2) What are the main hydrologic factors that affect these subsurficial stresses? We utilize daily hydrologic load arrays from several filling and operational scenarios to derive Coulomb stresses on optimal planes in a 3D elastic half-space to answer (1), and we investigate the relationships between both load area density and starting reservoir water levels with Coulomb stress results to answer (2). The results from this work will allow water managers to gain a deeper understanding of how different changes in reservoir inflow/outflow regimes affect subsurficial stresses within the study area. Knowledge of these stress changes is important so that the potential for triggered seismic events can be better understood. The first section of this article provides a broad introduction to hydrologic loading and its effects. It expands a bit on Coulomb stress changes as caused from these loads and discusses reservoir triggered seismicity. The second section describes the study area for this research and then describes the data and methods utilized within this paper. The third section highlights the results of this work and provides some interesting discussion based upon those results. The last section provides some conclusive remarks and highlights the unique results and key findings of this work.

## Materials and methods

### Study area

**Fig. 1:**
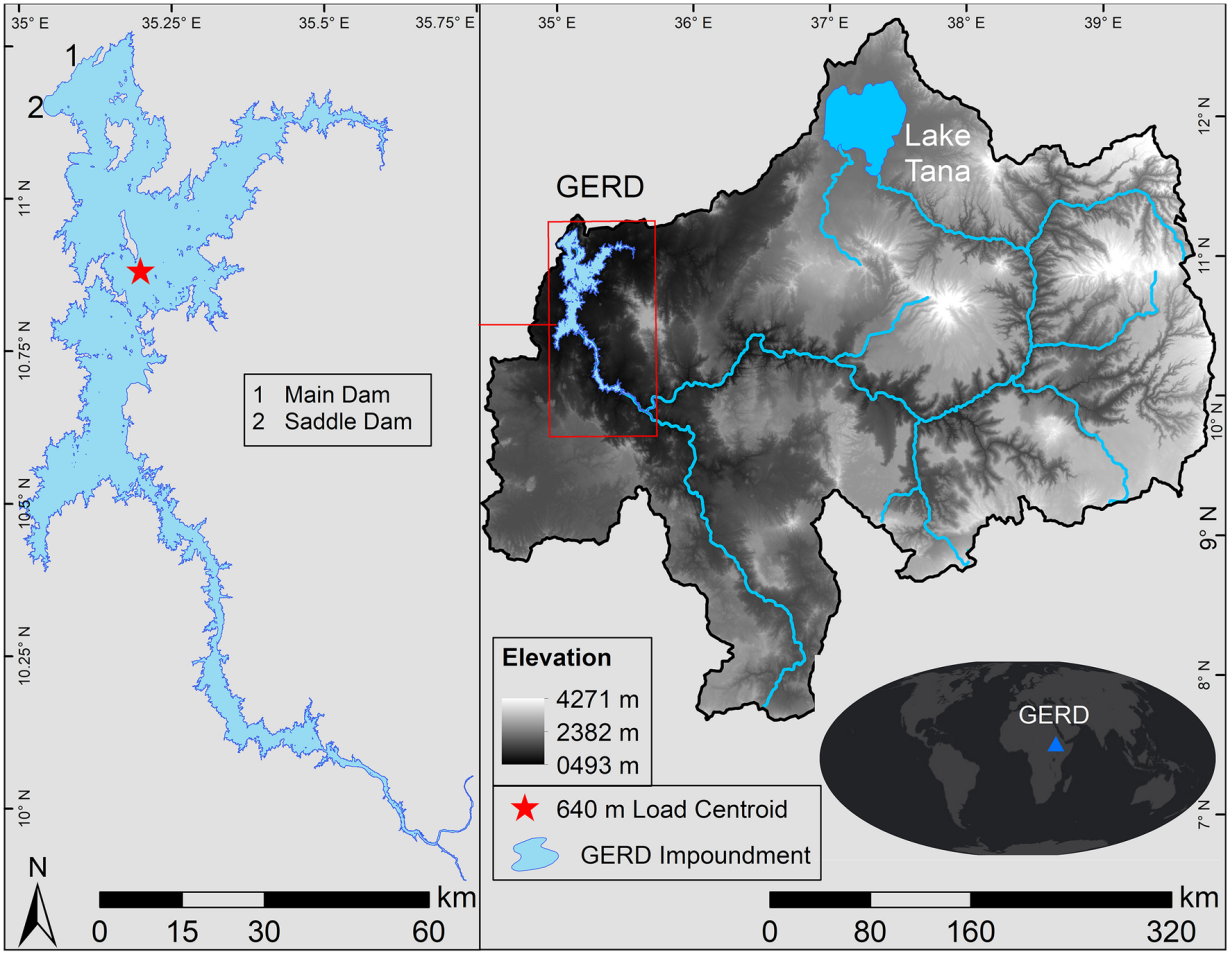
Overview of the GERD study area. The right plot shows the elevation of the Upper Blue Nile River Basin as well as the general location of the GERD impoundment. Plotted on the left is the GERD’s areal extent when full as well as the weighted hydrologic load centroid from the full impoundment. The main and saddle dams are labeled in the left plot. Reprinted from Madson and Sheng (2020)

The Grand Ethiopian Renaissance Dam (GERD) is located in Ethiopia on the Blue Nile several kilometers upstream from Ethiopia's border with Sudan and is slated for completion within the next few years (Abtew and Dessu 2019; Zhang et al. 2016). The GERD build site was one of four initially identified in the 1960s during a U.S. Bureau of Reclamation survey (Reclamation 1964). The location of the main dam infrastructure is plotted in Fig. 1 and is near the pour point of the Upper Blue Nile Basin. The Blue Nile itself originates at Lake Tana and drains the notable Ethiopian Highlands into the GERD impoundment and further towards the confluence with the White Nile. Work on the dam began in 2011 and will be the largest dam on the continent upon its completion. The GERD project consists of a 150 m tall and 1800 m long concrete main dam along with a saddle dam that is ~ 50 m tall and 5 km in length. The saddle dam increases the reservoir's water level to ~ 640 m (Ahmed and Elsanabary 2015; Mulat et al. 2018; Sharaky et al. 2017). The Blue Nile Basin accounts for about 58–62% of the entire Nile River water supply (Liersch et al. 2017). Flow data from the National Meteorological Agency of Ethiopia for the Blue Nile at the Sudanese/Ethiopian border have a historical mean annual flow of ~ 50 Gt where ~ 80% of the inflow occurs in July through October (Abtew and Dessu 2019; Abtew et al. 2009; Melesse et al. 2014).

Due to its size, the large reservoir behind the GERD impoundment will have several different impacts. For example, the annual discharge curve for the Blue Nile will be transformed due to the construction of the GERD and the notable reservoir that will accumulate behind the dam. The large capacity of the reservoir will allow for a uniform outflow over the entirety of the year. This will increase hydrologic stability by reducing the quantity of extreme Blue Nile flow events (low and high flows) (Liersch et al. 2017). There will likely be a decrease in the region’s hydrologic uncertainty owing to the reduction of drought and flood events. The GERD project is not the only major impoundment of the Nile River or the Blue Nile River. That said, the GERD’s upstream location in combination with the notable storage of the impoundment will likely have an effect on hydrologic projects further downstream. To that end, the Rosaries, Sennar, and Aswan High dams (all downstream of the GERD) will likely need to modify their outflow release operations so that Sudanese agricultural water supplies will be maintained (Wheeler et al. 2016). Further, the filling and operation of the GERD impoundment will likely affect downstream hydrologic power generation. The extent of these effects will be directly related to the operational and filling strategies that are decided upon by the GERD water managers (Beyene 2013; Sharaky et al. 2017; Wheeler et al. 2016).

### Impoundment plans, operational scenarios, and centroids

The initial filling plan for the GERD is not yet known. That said, this work utilizes filling strategies described in Mulat et al. (2018) and Liersch et al. (2017) to derive input water load calculations used in our initial impoundment stress modeling. Mulat et al. (2018) utilized natural inflow rates from 1973–1978 to derive an 80-month impoundment strategy. In this strategy the mean yearly inflow is around 0.5% larger than 1961–2002s mean yearly inflow of around 50 Gt, where the yearly outflow rate is never below ~ 30 Gt. This work utilizes monthly water levels from Mulat et al. (2018) to derive filling scenario *M1*. We point the reader to (Madson and Sheng 2020) for an in-depth look at how we derived the *M1* filling scenario. Liersch et al. (2017) derived monthly actual evapotranspiration (ET), precipitation, inflow, outflow, and seepage at the site of the GERD from January 1961 to December 1999. We derived monthly mean inflow datasets using three different categories of water years (average wet: from 1961–1981, average: from 1961–1999, and average dry: from 1981–1999) from the previously mentioned monthly hydrologic variables. The inflow rates for each of the three categories were utilized to derive monthly outflow rates based on a percentage value from the inflow (i.e. from 5 to 90% and at 5% intervals). For example, a 5% outflow value implies that 5% of the hydrologic inflow into the impoundment flows out, which equates to 95% storage. Reservoir storage was derived for the 18 different percentages of outflow rates for each of the three different categories of water years, and these monthly storage values were used to derive daily reservoir water elevations for all of the impoundment plans. These impoundment strategies are named *AW5–AW90*, *A5–A90*, and *AD5–AD90* (average wet, average, and average dry). Again, we point the reader to (Madson and Sheng 2020) for an in-depth look at how we derived these different filling scenarios. The water level arrays for these 55 unique filling scenarios (54 derived from Liersch et al. (2017) and one scenario derived from Mulat et al. (2018)) were utilized to calculate the impoundment loading grids which are used as the main inputs into our stress model as described in the following subsection.

The post-impoundment annual operational plans for the GERD are not yet known. That said, this work focuses its seasonal stress modeling on the five operational scenarios as discussed in Liersch et al. (2017). We name these five different operational strategies *L1–L5* and point the reader to (Madson and Sheng 2020) for a more detailed definition. These annual operational scenarios consist of monthly inflow, outflow, actual ET, seepage, and precipitation from January of 1961 to the end of December in 1999 (39 years). We derived two temporally different seasonal operation plans from these values for the five operational strategies. The first of these two temporally different datasets is comprised of one year of water storage changes and is derived from the individual months' mean values within the full multi-decadal operational dataset. In contrast, the second operational scenario is merely the entire 39-year monthly dataset. These two temporally different datasets were utilized to derive the annual hydrologic load arrays that are used as the main inputs into the stress model as described in the following subsection. We point the reader to (Madson and Sheng 2020) for more information on these scenarios and how they were derived.

Marked changes in hydrologic loads during impoundment and seasonal operations cause notable variations in the location of the weighted load centroids. These centroids mark the location of the maximum load for any given water level and are an important variable with respect to spatiotemporal changes in the stresses applied on the underlying rocks. The motion of the load centroid can be thought of as a proxy for the changes in the location of where the maximum stresses are applied on the Earth's crust. The load grids for each water level of the impoundment (500–640 m) from (Madson and Sheng 2020) were used to calculate the individual load centroids using a weighted mean center algorithm where the weight of the cell is assigned the water level at that location. The daily water level values for the 55 initial impoundment plans from the same study were employed to calculate the accumulated annual load centroid motion for each filling scenario. The weighted load centroid location for each daily reservoir level in the filling scenario was linearly interpolated using the two surrounding water levels’ weighted load center and the fractional part of the daily water level value. The distances between these daily load centroid locations were then accumulated for each 365-day period for all of the 55 different impoundment scenarios. The total accumulated weighted load centroid motion for each impoundment scenario was also derived. Similarly, the daily-accumulated annual weighted load centroid motion was calculated for both of the two temporally different monthly datasets (the single year and the entire 39-year) for the five separate operational strategies discussed in the previous paragraph.

### Coulomb stress

The previously described hydrologic load arrays from (Madson and Sheng 2020) were used to calculate the Coulomb stress on an optimal plane for each full-resolution cell (~ 30 m × ~ 30 m) in the array at 1 km depth increments from the surface (0 km) down to 25 km. We followed the method outlined in Liu and Zoback (1992) to derive the stress fields from the hydrologic load changes from the different impoundment and operational scenarios. All calculations were undertaken in an elastic half-space with a model domain of 300 km × 300 km × 25 km and at the full cell resolution. The horizontal dimensions of the model domain were selected such that regions with marked Coulomb stresses from the full impoundment would fall within the domain. For the subsequent calculations, both the first and second Lame’s parameters were set to 1, which assumes a Poisson ratio of 0.25. The final six stress tensor fields from the hydrologic loads were derived from their vertical [Boussinesq (Jeffreys 1924)] and horizontal [Cerruti (Love 1906)] stress components, per (Liu and Zoback 1992). To this end, altered scripts from (Styron and Hetland 2015) were utilized to calculate both of the horizontal and vertical components. The vertical stress component was derived by convolving the Boussinesq solutions as Green’s functions with the full hydrologic load array (Liu and Zoback 1992). We utilized a water density of 1000 kg/m^3^ and a gravitational constant of 9.81 m/s^2^ and took advantage of the superposition theorem to do the convolutions in the Fourier domain to speed up the calculations. The horizontal component consists of the two sets of stress fields from both the *x* and *y* horizontal surface tractions brought on by the vertical point load arrays on the half-space surface as calculated above. The two horizontal stress components were calculated by convolving the Cerruti solutions as Green's functions with their respective *x* or *y* loading function as derived from the appropriate surface traction for the full hydrologic load array (Liu and Zoback 1992). Similar to the above vertical component, we utilized a water density of 1000 kg/m^3^ and a gravitational force of 9.81 m/s^2^ and took advantage of the superposition theorem to do the *x* and *y* horizontal convolutions in the Fourier domain. Lastly, the stress fields from the vertical loading component and the two *x* and *y* horizontal components were summed to derive the final stress tensor field for each water level in the GERD impoundment and operational scenarios.

Next, the angle of an optimally oriented fault plane was calculated for each cell in the grid by following (Sibson 1974) while utilizing a friction coefficient of 0.6. This is the angle at which fault activation requires the lowest ratio of principal stress (Sibson 1985). The stress tensor arrays as calculated above were then utilized to determine the strike and dip angles of the optimally oriented fault plane for each cell in the grid. Next, the stress tensor arrays and the strike and dip angles for the optimally oriented planes were utilized to derive the shear stress by taking the maximum value between the along-strike and down-dip shear stresses. The normal stress on the optimal plane was then determined with the same stress tensor along with the plane's orientation (strike and dip angles). Lastly, the abovementioned shear and normal stresses were used to calculate the effective Coulomb stress changes following the equations in King et al. (1994). A friction coefficient of 0.6 was used throughout and pore pressure was ignored (set to zero) in the calculations. Pore pressure was neglected to solely focus on the static stress effects from the changing hydrologic load, and not the reduction in the optimal fault planes’ stability from the increased pore pressure and the subsequent reduction in frictional stresses caused by the diffusion of water into the underlying rock. We note that the friction coefficient utilized within our calculations (0.6) is somewhat conservative and allows for increased estimates of stability, and that a reduction in this parameter would have a destabilizing effect on our calculations of Coulomb stress. Further, the absence of the pore pressure parameter underestimates the results from our final Coulomb stress computations and that its inclusion into the calculation would further increase the overall instability (increased Coulomb stress values) of the optimal planes. The Coulomb stress model described herein consists of custom python scripts that follow the methodology as outlined above, and these scripts are based on work from (Styron and Hetland 2015).

The effective Coulomb stress for each water level of the full impoundment (500–640 m) in one-meter increments was derived following the workflow as explained in the preceding paragraph. The Coulomb stress for each load array was also calculated where the reservoir water elevation from the initial input water load file was the reservoir water elevation used to derive the surface area plus an extra 1 m of water elevation (501–641 m). This process was undertaken so that the Coulomb stress for each daily reservoir level in the filling scenarios could be linearly interpolated using the stress arrays of the two surrounding water levels and the fractional part of the daily water level value. For example, we derived the Coulomb stress arrays by first using the cells from the areal extent at the 620 m water level, and second by utilizing those same cells plus a water level increase of 1 m (i.e. 621 m). This procedure allows for the calculation of the daily Coulomb stress arrays as derived from the sub-meter changes in water level (for example, at 0.001 intervals between 620 and 621 m) without the need to derive the computationally expensive stress calculations over thousands of different iterations. The reservoir hydrologic load arrays for the 55 unique filling plans as described in Sect. Impoundment plans, operational scenarios, and centroids and the abovementioned stress arrays were utilized to derive the daily-accumulated Coulomb stresses for each of the GERD impoundment scenarios. Lastly, the two temporally different seasonal Coulomb stress arrays were derived (as described in the previous section). The mean annual scenarios were started on the first day of the first month where storage rate was a positive value (i.e. *L1*,* L2*,* L3*, and *L5*: July 1 and *L4*: June 1). Similarly, the yearly seasonal plans from the full operational dataset were started on the first date with a positive water storage value. These seasonal stress arrays were derived using input load arrays calculated from the difference between the hydrologic loads at the beginning of the annual operational season and at the peak of the season.

## Results and discussion

### Initial impoundment

The results and discussion herein focus on 22 different impoundment scenarios (AW45–AW75, A45–A75, AD45–AD75, and M1) because of the increased filling times and the lower levels of accumulated annual outflow at both of the upper and lower end of the percentage-based impoundment scenarios (see Madson and Sheng 2020). The former is important for both the time and operation of the reservoir water management and the latter plays a role in negating human impacts downstream. The filling time is also a meaningful parameter with respect to potential RTS where faster impoundments denote increased shear and normal stresses within a shorter time span while longer filling times may slow the delayed response to subsurficial draining which can cause an increase of the diffusive pore pressure through and within the underlying rock strata. We note that the impoundment scenarios each started on January 1 and nearly the first half of the first year in each filling plan displayed very little to no impoundment.

We were unable to locate regional seismogenic fault models in the area of the GERD impoundment due to a lack of available data, and, as such, were not able to derive Coulomb stress on known faults. Instead, we focused on the Coulomb stresses applied on optimally oriented fault planes within our study area. The workflow of the general research methodology is presented in Online Resource 1. Further results and discussion on Coulomb stresses from the GERD impoundment and operational scenarios are based on the stress tensors as calculated on optimal faults within the region, and we point the reader to Sect. Coulomb Stress for the overview of these calculations. That said, the stresses computed herein would likely be different if calculated on the actual seismogenic structures and would be dependent on their depth, location, and orientation with respect to the impoundment. Lastly, in the discussion that follows we are suggesting operational and filling strategies that are based on the results from these optimally oriented planes, and that these suggestions are speculative in nature. Further, the Coulomb stress changes skew towards higher values due to the fact that the stress changes are calculated on idealized fault planes. If there are seismogenic faults within the study area, they may not be oriented optimally towards the hydrologic induced stresses. Further, it is not certain that the GERD's hydrologic load changes will actually trigger local seismicity. The occurrence of RTS is dependent on if there are critically stressed faults present within the study area, and that the changes in shear and normal stresses along with the potential subsurficial pore pressure increase is enough to decrease the stability of the seismogenic faults beyond their failure point.

**Fig. 2:**
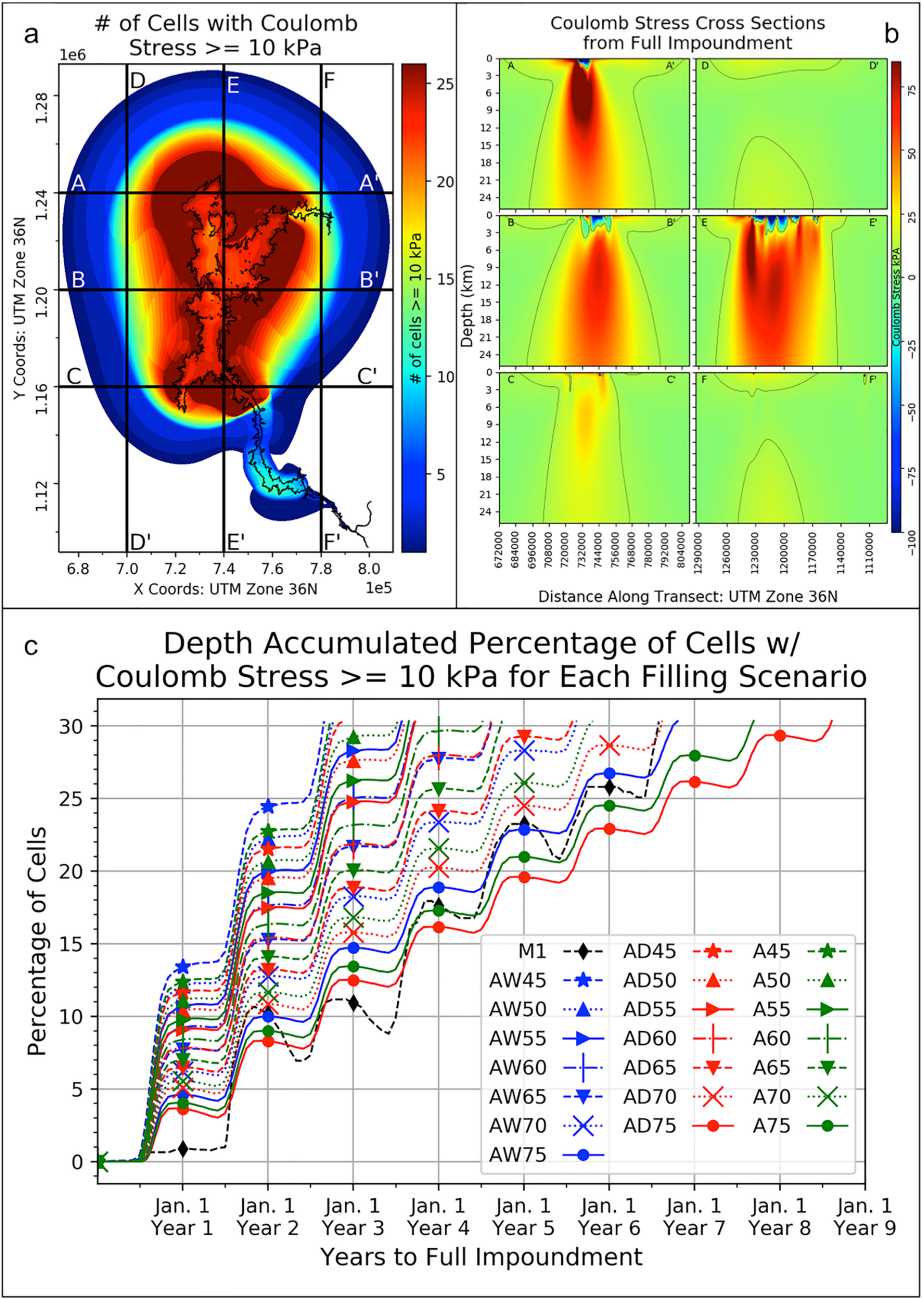
Number of cells with Coulomb stresses ≥ 10 kPa (**a**), Coulomb stress cross sections (**b**), and percentage of cells with a Coulomb stress ≥ 10 kPa (**c**) for 22 different filling scenarios. Vertical and horizontal lines in **a** denote cross section locations in **b**. Contour lines in **b** denote the location of the 10 kPa Coulomb stress regions. Cross sections are from west to east (e.g. A–A′) and north to south (e.g. D–D′). The model depth is from the surface (0 km) down to 25 km. The areal extent of the reservoir for the full impoundment (500–640 m) is plotted as the black polygon. Cell values at each depth in the model with Coulomb stresses ≥ 10 kPa are summed and then divided by the total number of grid cells (**c**). This is done for each day in the filling strategy. Temporal variations in accumulated Coulomb stress as caused by the different impoundment scenarios are apparent

The maximum subsurficial Coulomb stress derived on optimally oriented fault planes for the entire GERD impoundment as calculated from the datasets and methodologies explained in Sect. Materials and methods is ~ 186 kPa. We exclude the surficial Coulomb stresses to determine this maximum value and note that this maximum stress occurs at a depth of 1 km. The maximum Coulomb stress values range from ~ 1100 kPa at the surface of the model down to ~ 57 kPa at a depth of 25 km. To show the spatial extent of non-negligible stresses from the full impoundment we calculated the number of cells at each depth that have a Coulomb stress value ≥ 10 kPa. Coulomb stress increases in excess of 10 kPa are considered to be the threshold at which seismicity is affected (Reasenberg and Simpson 1992; Stein 1999). These depth-accumulated values are plotted in Fig. 2a along with six example cross sections of the Coulomb stress fields (Fig. 2b) for the full GERD impoundment (500–640 m). We provide the Coulomb, normal, and shear stress arrays from the full GERD impoundment and for each depth (0 km to 25 m) in our model in Online Resource 8. The location of the maximum Coulomb stress for each depth is plotted in the animation as a white cross and the contour lines denote the location of the 10 kPa Coulomb stress regions. The darkest red regions in Fig. 2a show that the area immediately adjacent to the main body of the full impoundment incurs Coulomb stresses ≥ 10 kPa at all depths in our model (0–25 km), and a closer look at cross sections for A–A′, B–B′, C–C′, and E–E′ in Fig. 2b shows detailed views along the depth axis in which this is the case. We reiterate that these are Coulomb stress arrays on idealized fault planes within our model regime.

To appropriately investigate and compare the timing of the stress state changes for the 22 filling scenarios we derived the daily depth-accumulated percentage of cells that have a Coulomb stress ≥ 10 kPa. These percentages include the Coulomb stresses at the surface of the model (0 km) and we note that the temporal pattern for the individual filling scenarios would be the same as if they were derived from all depths sans the surface (albeit with slightly lower percentages as caused by the removal of the surficial stress cells). The daily depth-accumulated percentage of cells that have a Coulomb stress ≥ 10 kPa for each of the 22 filling scenarios is plotted in Fig. 2c. This analysis allows for a direct comparison of the Coulomb stresses within the model domain and the timing of these results from the many different impoundment scenarios. The percentage of cells reaches a ~ 30% maximum for every filling scenario because of the fact that the maximum Coulomb stress at the end of the line plots is from the full impoundment (500–640 m) regardless of the filling strategy. This implies that the depth-accumulated plot in Fig. 2a would be the same for the 22 different impoundment plans as the volume and extent from the full impoundment is the same regardless of the filling strategy used.

Lower percentages denote periods in the impoundment strategies where there is a reduced amount of modeled cells that are exposed to Coulomb stresses greater than the 10 kPa threshold. Although the magnitude of the overall depth-accumulated percentage of cells with a Coulomb stress ≥ 10 kPa is the same for each filling scenario (~ 30%), it is the timing of these accumulations that is markedly different. Here, we note that 13 of the 22 different impoundment scenarios (*A45–60*, *AW45–65*, *AD45–60*) have about 50% of their total depth-accumulated cells ≥ 10 kPa within the first two years of the filling scenario. However, if the depth-accumulated percentage of cells is divided by the total time to full impoundment for every plan shown in Fig. 2c, we discover that the five strategies with the smallest amount of daily cells that meet the 10 kPa stress threshold are *AD75* (0.0096), *A75* (0.0107), *AW75* (0.0121), *AD70* (0.0124), and *M1* (0.0125%/day). Each of these five scenarios has a depth-accumulated cell-per-day total equating to an areal extent of 65.06, 72.79, 82.36, 84.54, and 85.06 km^2^/day, respectively. In comparison, the bottom-five scenarios (*AW45*, *A45*, *AW50*, *AD45*, and *A50*) each have a depth-accumulated cell-per-day total equating to an areal extent of 210.73, 205.02, 201.98, 193.75, and 159.07 km^2^/day, respectively. That said, these results show the five filling scenarios selected to reduce the daily depth-accumulated areal extent exposed to Coulomb stresses ≥ 10 kPa (based on optimally oriented faults) out of the 22 impoundment strategies investigated. It is important to note that the highest and lowest rates are from the five shortest and longest impoundment scenarios as the total accumulated percentage of cells is the same for each of the filling scenarios. We reiterate that the overall Coulomb stress from the entire GERD impoundment will very likely not happen during a single filling season, but instead it will be spread over the particular impoundment strategy that is eventually decided upon by the GERD water managers. That said, Fig. 2c shows how the Coulomb stresses ≥ 10 kPa accumulate over 22 different impoundment plans at the GERD.

To further investigate the differences between the 22 different filling scenarios we derived the annual depth-accumulated distance change in the location of the maximum daily Coulomb stress. Simply put, the location of the maximum Coulomb stress for each of the 26 depths in our model was determined for each day during the 22 different impoundment strategies. The day-to-day change in the location of these max stresses was then determined at all depths. Lastly, the motion at each depth for each day was accumulated and these distances for every individual year in the filling scenario were summed. These depth-accumulated daily maximum Coulomb stress distances are plotted in Online Resource 2. These plots provide meaningful information as to the timing and motion changes of large Coulomb stresses brought on by the individual impoundment scenarios. They can act as a proxy for the comparison of the spatiotemporally varying stress changes imposed on the surrounding lithosphere from the individual filling scenarios. It is evident that the bulk of the maximum Coulomb stress motion occurs during the second-half of each year in the impoundment, and we attribute this to the marked seasonal hydrologic regime at the GERD site where the bulk of the inflow occurs during only a handful of months in the year. We note that the filling scenarios plotted in Online Resource 2 with lower end-of-the-year values denote impoundment strategies with a reduced amount of modeled area exposed to the maximum Coulomb stresses. In a sense, these lower accumulated distances can decrease the areal range where notable stresses are applied on potential seismogenic faults, and, in turn can also decrease the likelihood for these load-induced stresses to increase fault instability. The more these maximum Coulomb stresses migrate during the impoundment, the more areal extent is covered by these marked stress states and the more likely they are to interact with and push the optimally oriented faults to failure.

**Fig. 3:**
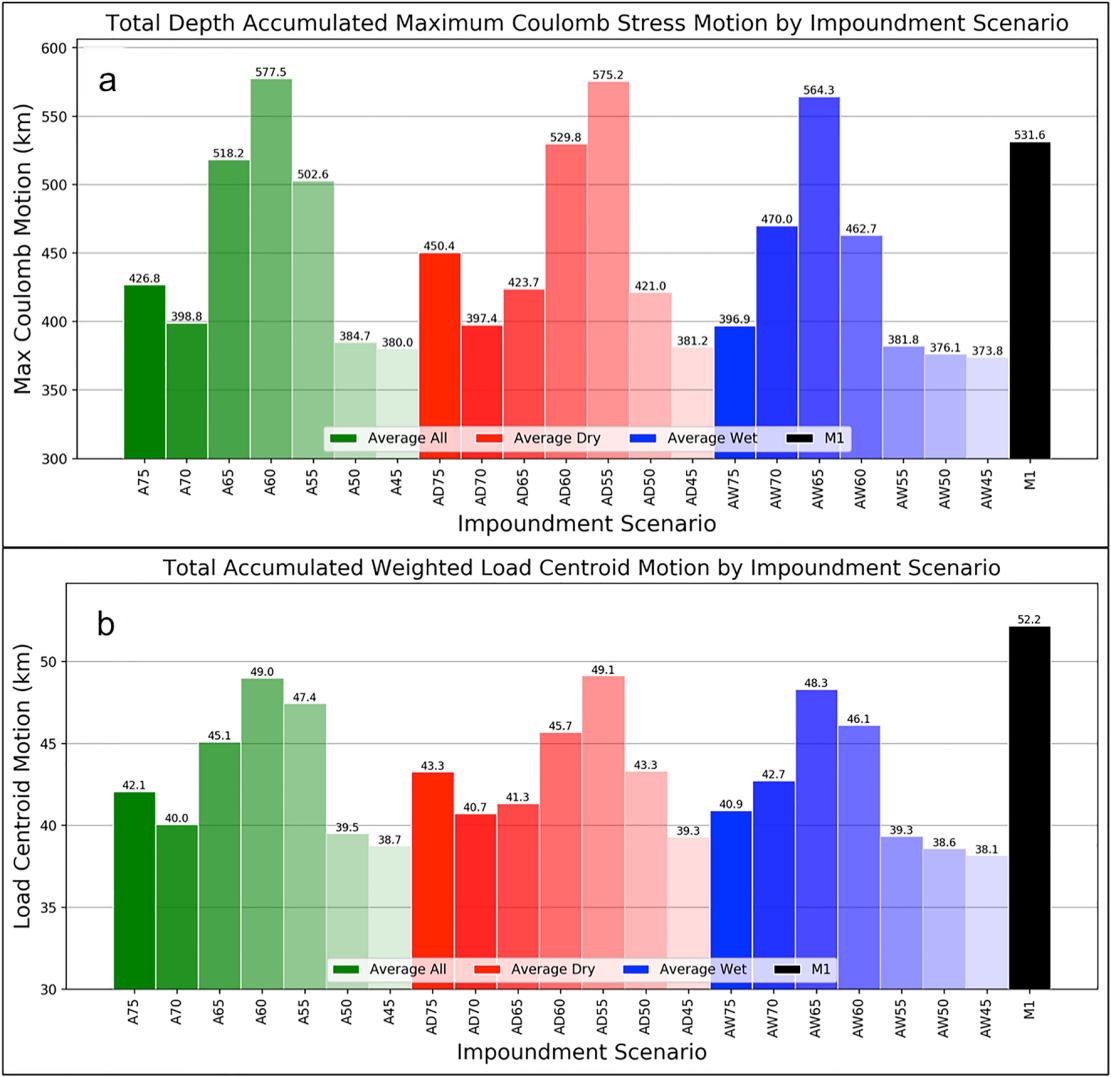
Total depth-accumulated movement of the maximum Coulomb stress cell (**a**) and total accumulated motion of the weighted hydrologic load centroid (**b**) for 22 different filling scenarios. **a** Is the summation of the depth-accumulated motion of the maximum Coulomb stress as calculated on an array of optimally oriented faults and **b** is the accumulated motion of the weighted load centroid. Here, lower numbers denote filling scenarios where there is a reduced amount of sub-surface area exposed to the maximum Coulomb stresses (**a**) and where there is a reduced motion in the location of the weighted load centroid (**b**)

To better explore the differences between the 22 different impoundment strategies we sum the yearly distances for each filling scenario and plot the accumulation of these annual max Coulomb stress motions in Fig. 3a. It is evident that the shorter impoundment scenarios (*A45*, *AD45*, and *AW45*) have the lowest accumulated max Coulomb stress motion. Again, if the max Coulomb stress motion is divided by the individual scenarios' filling time as shown in Fig. 3a, we discover that the five impoundment plans with the smallest daily motion of max Coulomb stress cells are *AD75* (0.14), *A75* (0.15), *AW75* (0.16), *AD70* (0.16), and *A70* (0.19 km/day). The five impoundment plans with the greatest daily motion of max Coulomb stress cells are *AD55* (0.42), *AW45* (0.39), *A55* (0.38), *A45* (0.38), and *AW50* (0.37 km/day). That said, these results show the five filling scenarios selected to reduce the depth-accumulated areal extent exposed to the maximum Coulomb stress (based on optimally oriented faults) out of the 22 impoundment strategies we investigated.

In a similar comparison, we derived the motion of the weighted hydrologic load centroid following the methodology outlined at the end of Sect. Impoundment plans, operational scenarios, and centroids. The total accumulated weighted load centroid motion for each impoundment scenario is plotted in Fig. 3b. The total centroid motion was divided by the filling time for each scenario, and, similar to the results in the last paragraph, we find that the five plans with the smallest daily centroid motion are *AD75* (14), *A75* (15), *AW75* (17), *AD70* (17), and *A70* (19 m/day). In comparison, the five scenarios with the highest daily centroid motion are *AW45* (39), *A45* (39), *AW50* (38), *AD45* (37), and *AD55* (36 m/day). We note that the scenarios with the lowest hydrologic load centroid rates are similar to their max Coulomb stress motion rate counterparts as mentioned in the previous paragraph. Further, the examination of Fig. 3 highlights the similar pattern between the motion rates of the load centroids and max Coulomb stresses. As such, it would appear that weighted hydrologic load centroid motion is a good proxy for maximum Coulomb stress cell motion on optimally oriented fault planes, and vice versa. This proxy calculation is much less computationally intensive than deriving the actual distance of the maximum Coulomb stress changes. However, we note that these results are only a proxy for stress motion and not the actual stress change itself.

Upon comparison of the above stress vector and hydrologic load centroids for the 22 unique impoundment plans we note that the more meaningful results are with the depth-accumulated maximum Coulomb motion and the load centroid movements. This is because the results from the percent Coulomb stress analysis have a constant value with which to derive their rates (i.e. The rate calculations are based on the same value due to the fact that the total percentage of Coulomb stress cells ≥ 10 kPa is the same for each impoundment scenario). That said, the five filling strategies that provide for the lowest maximum Coulomb stress motion and the lowest weighted hydrologic load centroid motion per impoundment are *AD75*, *A75*, *AW75*, *AD70*, and *A70*. We note that these five impoundment strategies are the longest-running, non-*M1* strategies of the 22 investigated. Impoundment scenario *M1* is not included (even though its filling time is longer than *A70*’s) as its accumulated maximum Coulomb stress and load centroid distance per unit time is notably higher than the abovementioned scenarios (as evidenced in Fig. 3a, b). This stems from the different input hydrologic variables utilized to derive the load input arrays (daily water level, volume, and areal extent changes). Upon inspection of Figure S7 from Madson and Sheng (2020) it becomes evident that during the *M1* filling scenario there exists a seasonal period of non-negligible negative storage in all but the first year of the impoundment scenario. In some cases this seasonal negative storage value equates to ~ 5 Gt (i.e. during year 2–3 of the *M1* scenario). This is in direct comparison to all of the other impoundment scenarios (Figures S3–S6 from Madson and Sheng 2020) where there are notably fewer seasons in the filling scenarios where negative water storage occurs. Further, when negative storage values do happen, they are far lower than the values that occur in the *M1* filling scenario. This is the reason that we see larger values in the motion of the depth-accumulated maximum Coulomb stress location as well as the weighted hydrologic load centroid per unit time for the *M1* scenario as compared to the *AD75*, *A75*, *AW75*, *AD70*, and *A70* impoundment scenarios. This implies that an increase in total accumulated daily reservoir storage change (both positive and negative) will cause an increase in the motion of both the depth-accumulated maximum Coulomb stress location as well as the weighted hydrologic load centroid. This makes intuitive sense seeing as how the increased change in the reservoir’s hydrologic load will alter the location of the load's centroid as well as the maximum Coulomb stress.

### Seasonal operations

The notable differences between the operational scenarios investigated in this study show how large the effect reservoir operations have on annual water load variations (Madson and Sheng 2020). These different hydrologic load fluctuations will in turn control the magnitude and spatiotemporal changes of the load-induced Coulomb stresses in the region during any given operational year. The seasonal stress model runs utilize a 622 m starting reservoir water level and were started on the first day with a positive reservoir water storage value (i.e. *L1*,* L2*,* L3*, and *L5*: July 1 and *L4*: June 1). This particular starting water level was selected as Mulat and Moges (2014) specify it as the lowest operating water level for the GERD impoundment. However, a few other researchers state the lowest operating water level is 590 m (IPoE 2013; Jameel 2014). That said, our stress models were run with both 590 and the 622 m as the initial water level starting elevation. It is important to note that the total water storage change is equal for each of the two water level starts and that the seasonal water extent and level fluctuations are both larger when a starting water level of 590 m is utilized. The maximum subsurficial Coulomb stresses derived on optimally oriented fault planes for both the 590 and 622 m mean annual operational scenarios (*L1*–*L5*) as calculated from the methods and datasets described in Sect. Materials and methods are 44.7, 44.9, 44.3, 39.1, 34.7 kPa and 26.0, 26.2, 25.8, 22.2, 19.4 kPa, respectively. The surficial Coulomb stresses were excluded to determine this maximum value and this maximum stress occurs at a depth of 1 km for each scenario. The maximum Coulomb stresses for operational scenarios *L1*–*L5* with a 590 m starting water level range from 87 to 20 kPa (*L1–L3*), 78 to 17 kPa (*L4*), and 69 to 15 kPa (*L5*) from the surface (0 km) down to the base of the model (25 km), respectively. In contrast, the maximum Coulomb stresses for operational scenarios *L1*–*L5* with a 622 m starting water level range from 53 to 16 kPa (*L1–L3*), 45 to 14 kPa (*L4*), and 41 to 12 kPa (*L5*) from the surface (0 km) down to the base of the model (25 km), respectively. The maximum Coulomb stresses for each of the five mean annual scenarios are around 18.7 (*L1* and *L2*), 18.5 (*L3*), 16.9 (*L4*), and 15.3 kPa (*L5*) larger in the 590 m water level start model runs when compared to the results from the 622 m water level start model runs. We attribute this difference to a larger water load per areal unit during the 590 m starting water level model runs. To that end, we note that the reservoir storage for each of the two starting water levels is the same, but the final extent of the surface water during the 590 m data runs is less than the 622 m water level start counterparts. This, of course, increases the water load per areal unit during the 590 m water level start model runs. This increased load density is the cause of the amplified subsurficial Coulomb stress changes for the 590 m model results.

Previous studies have derived seasonal Coulomb stress changes from annual hydrologic loading scenarios. In particular, Craig et al. (2017) examined the Coulomb stress changes for two faults within the New Madrid seismic zone. They found that the seasonal hydrologic changes altered the stress on the faults by around 1 kPa for each seasonal cycle. Further, Johnson et al. (2017) examined seasonal Coulomb stress changes on seismogenic faults in California from annual hydrologic changes (e.g. from snow, groundwater, surface water). They found that some faults could see peak-to-peak seasonal changes in Coulomb stresses of around 1.5 kPa. Their research has shown seismicity increases during increased hydrologic induced stress conditions and they have surmised that seismicity rates in the region are somewhat controlled by the area’s hydrologic loading and unloading regime. We note that our seasonal results show increased Coulomb stress changes than the two previously mentioned studies due to the much larger and more condensed volume of water as well as the fact that our model derives stress on idealized fault planes (as opposed to actual fault geometries that are likely not oriented in such an idealized manner). Further, recent work by Zhang et al. (2020) where they modeled Coulomb stress changes from reservoir loading and unloading in China has shown that stress changes on the order of 1–100 kPa were found ranging from the surface down to depths of 15 km. These stresses were modeled according to the typical fault geometries in the region and are more aligned with the results from within this research.

**Fig. 4:**
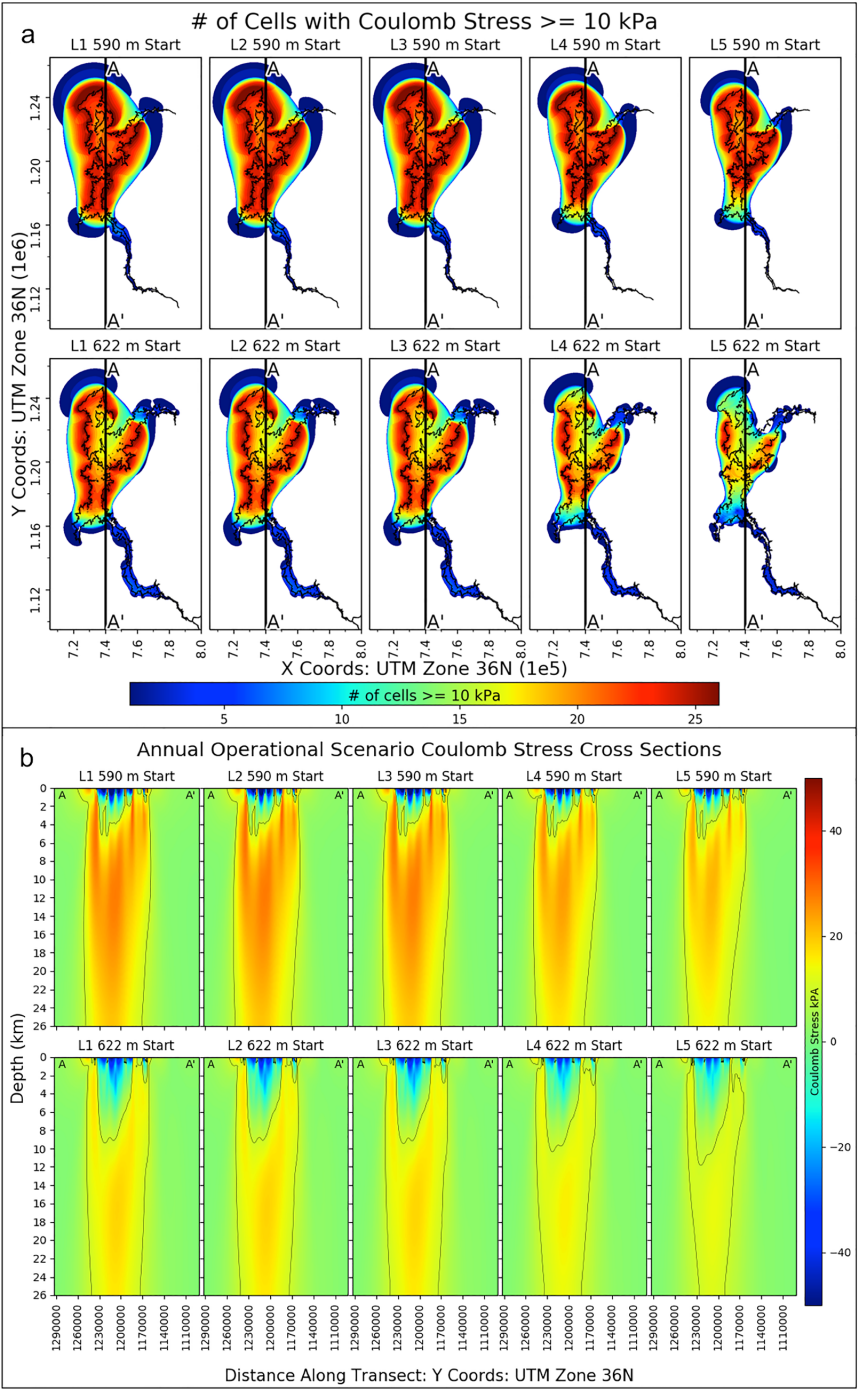
Depth-accumulated count of grid cells with Coulomb stresses ≥ 10 kPa (**a**) and Coulomb stress cross sections through the heart of the impoundment (b) for both starting water levels (590 m: top and 622 m: bottom) and each mean annual operational scenario (*L1*–*L5*). The vertical lines in **a** denote the cross section locations for the plots in **b**. The areal extent of the reservoir for the individual mean annual scenarios’ maximum water level is plotted as the black polygon in each subplot within (**a**). Contour lines in **b** denote the location of the 10 kPa Coulomb stress regions. Cross sections are from the north to south (e.g. A–A′) and their locations are plotted in **a**. The model depth is from the surface (0 km) down to 25 km

To show the spatial extent of non-negligible stresses from each operational scenario (and both starting water levels) we calculated the number of cells at each depth that have a Coulomb stress value ≥ 10 kPa. These depth-accumulated values are plotted in Fig. 4a along with an example cross section of the Coulomb stress fields (Fig. 4b) for operational scenarios *L1*–*L5*. The Coulomb, normal, and shear stress arrays from each of these operating strategies at the 590 and 622 m starting water levels for each depth (0 km to 25 m) in our model are provided in Online Resource 9–18. Again, we note that Coulomb stress increases in excess of 10 kPa are considered to be the threshold at which seismicity is affected (Reasenberg and Simpson 1992; Stein 1999). The darkest red regions in Fig. 4a show the areas immediately adjacent to the main body of the average seasonal impoundment that incurs Coulomb stresses ≥ 10 kPa at all depths in our model (0–25 km). Example cross sections (A–A′) through the heart of the reservoir for the five different mean annual operational scenarios and the two different water level starts are provided in Fig. 4b. These provide detailed views along the depth axis which allows for a comparison of subsurficial Coulomb stresses for each of the ten different mean annual operations. It is evident from the range of maximum Coulomb stresses per depth, the number of cells with Coulomb stresses ≥ 10 kPa (Fig. 4a), and the Coulomb cross sections (Fig. 4b) that the 590 m water level start scenarios have larger subsurficial stress regimes as compared to their 622 m water level start counterparts. Again, we reiterate that these are Coulomb stress arrays on idealized fault planes within our model regime.

The depth-accumulated percentage of cells that have a Coulomb stress ≥ 10 kPa was determined to appropriately investigate and compare the stress state changes for the two different mean annual operational scenarios (590 and 622 m start). These percentages include the Coulomb stresses at the surface of the model (0 km) and we note that the stress arrays are calculated from hydrologic loads based on the starting water level and the seasonal peak water level for each of the ten individual mean annual operational strategies. The total depth-accumulated percentage of cells that have a Coulomb stress ≥ 10 kPa for the 590 m and the 622 m starting water levels for each operational scenario are 10.8, 10.9, 10.6, 8.8, 7.2% and 9.1, 9.2, 9.0, 6.6, 4.5%, respectively. Lower percentages denote strategies where there is a reduced amount of area exposed to Coulomb stresses greater than the 10 kPa threshold. The scenarios for the 590 m starting water level have around 1.6–2.7% more depth-accumulated cells than their 622 m starting water level counterparts. This implies that all five mean annual operational scenarios (*L1*–*L5*) with a 590 m starting water level have an increased amount of optimally oriented fault cells with a Coulomb stress of at least 10 kPa when compared with the 622 m starting water level. The total hydrologic load applied for each corresponding scenario at both water level start dates is the same, but the areal extent at the peak load between the two starting elevations is notably different. The difference in the overall areal extent at which the load is distributed is mostly dependent on the starting water level of the scenario. Recall from a previous paragraph in this section that the 590 m water level start model runs have increased hydrologic loads per unit area as compared to their 622 m starting water level counterparts due to the fact that the final surface water extents for those data runs is smaller in comparison. In turn, this increases the water load per unit area for those data runs. The load per unit areal coverage for each of the five operational strategies for the 590 m starting water level is 20.55, 20.57, 20.38 18.97, and 17.17 ton/m^2^ as compared to 13.43 13.46, 13.29, 11.95, and 10.54 ton/m^2^ for the 622 m starting water level. The loads per areal unit during the 590 m model runs are nearly double that of their corresponding 622 m data runs. This is a notable difference and we attribute this as the cause for the increased amount of Coulomb stress for the 590 m starting water level scenarios.

The weighted hydrologic load motion was calculated following the methodology outlined at the end of Sect. Impoundment plans, operational scenarios, and centroids, and the annual accumulated weighted load centroid motion for the 590 and the 622 m starting water levels for each operational scenario are 14.96, 14.99, 14.81, 13.32, 11.72 km and 8.03, 8.05, 7.94, 6.98, 6.09 km, respectively. The motion of the hydrologic load centroids for each scenario is similar to their depth-accumulated Coulomb stress percentage counterparts listed in the previous paragraph. We attribute the larger load centroid motion of the 590 m data runs to the increased annual range of water extent and level changes as compared to the 622 m water level start. The seasonal amplitudes of water level and areal extent change for both the 590 and 622 m starting water levels for the five mean annual operational scenarios (*L1*–*L5*) are 27.3 (570.6), 27.4 (573.4), 27.1 (564.1), 23.9 (484.3), 21.2 m (421.4 km^2^) and 15.6 (475.3), 15.7 (477.8), 15.5 (469.5), 13.4 (399.0), 11.7 m (344.9 km^2^), respectively. These differences allow for the increased load centroid motion for the 590 m water level start scenarios.

The results from the previous few paragraphs highlight the importance of the initial and peak water levels for the mean annual operational scenarios. The Coulomb stresses from a more condensed seasonal reservoir load will be larger than the Coulomb stress as calculated from the same hydrologic load with less load per unit area. In contrast, for a given seasonal hydrologic load, a decrease in the load per unit area would reduce the overall Coulomb stresses on the optimal fault planes. We note that these are comparisons between five different mean annual scenarios, and we focus on the full 39-year operational dataset in subsequent paragraphs. These long-term scenarios are investigated to better understand the dissimilarities in Coulomb stress between differing seasonal amplitudes of load changes and initial water levels.

The maximum (and minimum) subsurficial Coulomb stresses for the most extreme annual amplitudes of water load change during the entire 39-year hydrologic dataset for operational strategies *L1*–*L5* are 67.51 (10.58), 60.76 (10.77), 54.80 (11.55), 75.91 (7.32), and 44.16 kPa (6.44 kPa), respectively. The seasonal maximum and minimum subsurficial Coulomb stress for these scenarios over the full 39-year model runs are 75.91 and 6.44 kPa, respectively. For context, the maximum subsurficial Coulomb stresses from the highest amplitude season in the entire 39-year operational dataset for all five scenarios are 36.30, 32.67, 29.46, 40.81, and 23.74% of the total maximum subsurficial Coulomb stresses brought on by the entire GERD impoundment. These notable ranges in Coulomb stresses further highlight the stark differences of the different hydrologic operational scenarios at the GERD. We note that the starting water level value for the full hydrologic operational model runs was set so that the reservoir water elevation during the entire multi-decadal hydrologic dataset never exceeded the 640 m maximum reservoir level value of the GERD. The start of a seasonal hydrologic cycle is defined as the very first date of positive water storage and the end of the seasonal hydrologic cycle as the first date where the reservoir storage begins to increase (i.e. the entire inflow and outflow curve of a season). In some cases, this means that seasonal cycles are not exactly 365 days long.

**Fig. 5:**
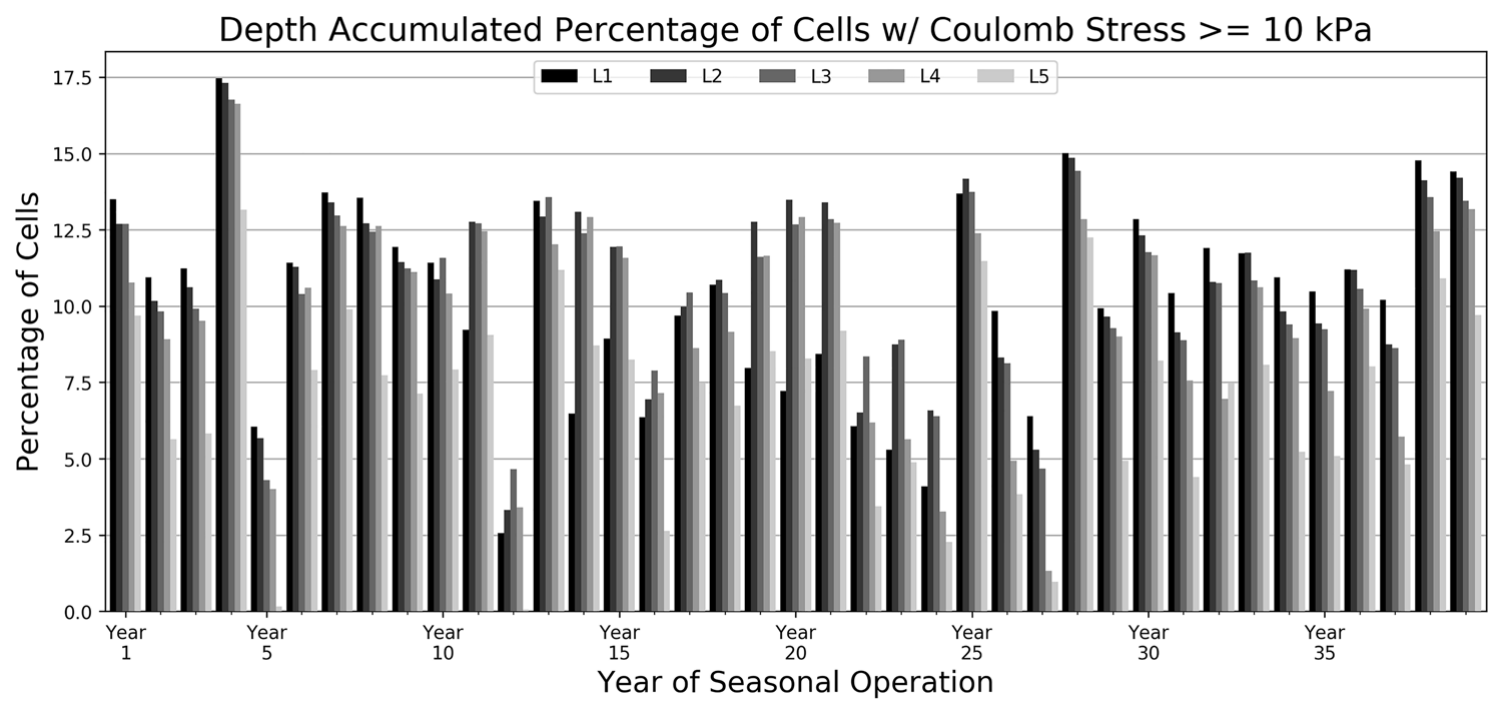
Depth-accumulated percentage of cells with a Coulomb stress ≥ 10 kPa for five different operational scenarios for the entire 39-year operational dataset. The Coulomb stress on optimally oriented fault planes was calculated from hydrologic loads based on the starting water level and the seasonal peak water level for each individual year

The depth-accumulated percentage of cells that have a Coulomb stress ≥ 10 kPa was determined to appropriately investigate and compare the seasonal stress state changes for the full 39-year operational scenarios (*L1*–*L5*). These percentages include the Coulomb stresses at the surface of the model (0 km) and the stress arrays are calculated from hydrologic loads based on the starting water level and the seasonal peak water level for each year in the full operational dataset. The season total depth-accumulated percentage of cells that have a Coulomb stress ≥ 10 kPa for each of the annual operational plans are plotted in Fig. 5. Lower percentages denote strategies where there is a reduced amount of modeled area exposed to Coulomb stresses greater than the 10 kPa threshold for that particular season. There are marked differences in the percentages of Coulomb stress cells between the five different scenarios in any given season and between each individual annual operation within the full dataset. We attribute these variations to the hydrologic load per unit area of the seasonal reservoir loads for each of the annual operations. We point the reader to Online Resource 3 that highlights the relationship between the seasonal load per unit area and the number of depth-accumulated Coulomb stress cells ≥ 10 kPa. This scatter plot shows that as the load per unit density increases so to does the amount of stress cells ≥ 10 kPa. This implies that a reduction in the seasonal hydrologic load per unit area will likely reduce the number of cells with notable Coulomb stresses. Further, due to the topography of the study area, we note that there is an overall decrease in the hydrologic load per unit area as the water level at the beginning of the operational season increases. This relationship is highlighted in Online Resource 4. It can be inferred that, typically, the higher the reservoir level is at the start of a given operational season the fewer cells will incur Coulomb stresses ≥ 10 kPa.

To better compare the differences between the five operational scenarios we determined the overall total percentage of Coulomb stress cells ≥ 10 kPa for the five different 39-year operational strategies. These percentages are plotted in Online Resource 5. Operational strategies *L1*, *L2*, and *L3* have the highest accumulated amount of cells followed by *L4* and then *L5*. These results follow a similar trend as previously discussed comparisons between strategies and are attributed to the overall differences in annual reservoir storage between the operational scenarios (Figure S8 from Madson and Sheng 2020).

The seasonal-accumulated daily weighted load centroid motion for the five operational scenarios for each of the 39 years in the GERD operational dataset is plotted in Online Resource 6. The motion of the load centroid can be thought of as a proxy for the changes in the location of where the maximum stresses are applied on the Earth’s crust. Similar to the results in Fig. 5, there are notable annual differences in the motion of the hydrologic load centroid for each scenario. Again, we attribute these marked variances to the different seasonal load per unit areas and the water levels at the start of each season. The relationship between the accumulated annual centroid motion and the reservoir water level at the beginning of the season is highlighted in Online Resource 7. It can be inferred that, typically, the higher the reservoir level is at the start of a given operational season, the less the weighted load centroid travels during that time period.

The comparisons of the stress vector and hydrologic load centroids for each annual operational scenario have shown that the water level at the beginning of the season as well as the seasonal reservoir load per unit area play a major role in the amount of stress applied on the surrounding lithosphere. We reiterate that the above discussion is based on the Coulomb stresses on optimally oriented fault planes and that we were unable to locate regional seismogenic fault models in the area of the GERD impoundment. That said, this investigation has laid the groundwork for future studies to examine Coulomb stress on known seismogenic faults and to explore the stress vector responses on different impoundment and operational scenarios to reduce the likelihood of triggered seismic events during reservoir filling and operational scenarios.

## Conclusions and recommendations

This work has provided a first look at the Coulomb stress and hydrologic load centroid movements as caused by several different modeled reservoir impoundment and operational plans for the GERD on the Blue Nile River. This research has helped to better understand the spatiotemporal dynamics and amplitudes of the hydrologic load-induced stresses within the GERD study area. These changes can have implications for induced seismicity in the region and water managers can apply these results to derive meaningful impoundment and reservoir operational scenarios. Hydrologic loads from several initial impoundment and reservoir operational scenarios were utilized to derive the subsurficial stress and load changes at the study site. We found that the main driver behind the stress and load centroid motion was the annual, accumulated daily reservoir storage change (both positive and negative) where an increased volume change caused an increase in the motion of both the depth-accumulated maximum Coulomb stress location as well as the weighted hydrologic load centroid. Further, we found that the variations in annual Coulomb stress changes were attributed to the hydrologic load per unit area of the seasonal reservoir loads for each of the annual operations, and, in part to the initial seasonal water level. The Coulomb stress from a more condensed seasonal reservoir load was larger than the Coulomb stress as calculated from the same hydrologic load with less load per unit area. In other words, a reduction in the seasonal hydrologic load per unit area or an increased initial seasonal water level would likely reduce both the number of cells with notable Coulomb stresses and the accumulated annual centroid motion. Future work entails acquiring seismogenic fault geometries in the region and applying our Coulomb stress models on those fault planes. The results from this work allow water managers to gain a deeper understanding of how different changes in reservoir inflow/outflow regimes affect subsurficial stresses within the study area. Knowledge of these stress changes is important to better understand the potential for triggered seismic events.

## Supplementary information

Below is the link to the electronic supplementary material.

Supplementary file1 (DOCX 6568 kb)

Supplementary file2 (GIF 21818 kb)

Supplementary file3 (GIF 19512 kb)

Supplementary file4 (GIF 19545 kb)

Supplementary file5 (GIF 19419 kb)

Supplementary file6 (GIF 18659 kb)

Supplementary file7 (GIF 17870 kb)

Supplementary file8 (GIF 19748 kb)

Supplementary file9 (GIF 19786 kb)

Supplementary file10 (GIF 19740 kb)

Supplementary file11 (GIF 19124 kb)

Supplementary file12 (GIF 18531 kb)

## Data Availability

The data utilized for this study are available at the following locations (1) JAXA ALOS AW3D30 DEM data (https://www.eorc.jaxa.jp/ALOS/en/aw3d30/index.htm) and (2) GERD hydrologic data (https://doi.org/10.3390/w9100728) and (https://doi.org/10.1016/j.ejrh.2018.02.006).
