# Supplementary material for: Coulomb stress analysis for several filling and operational scenarios at the Grand Ethiopian Renaissance Dam impoundment
Source: Environ Earth Sci. 2021 Mar 28;80(7):286. doi: 10.1007/s12665-021-09591-w (PMC8550559; doi:10.1007/s12665-021-09591-w)
Supplement: Supplementary file 1 — Supplementary file1 (DOCX 6568 kb) [file 12665_2021_9591_MOESM1_ESM.docx]

**Online Resource 1.** Research methodology flow chart


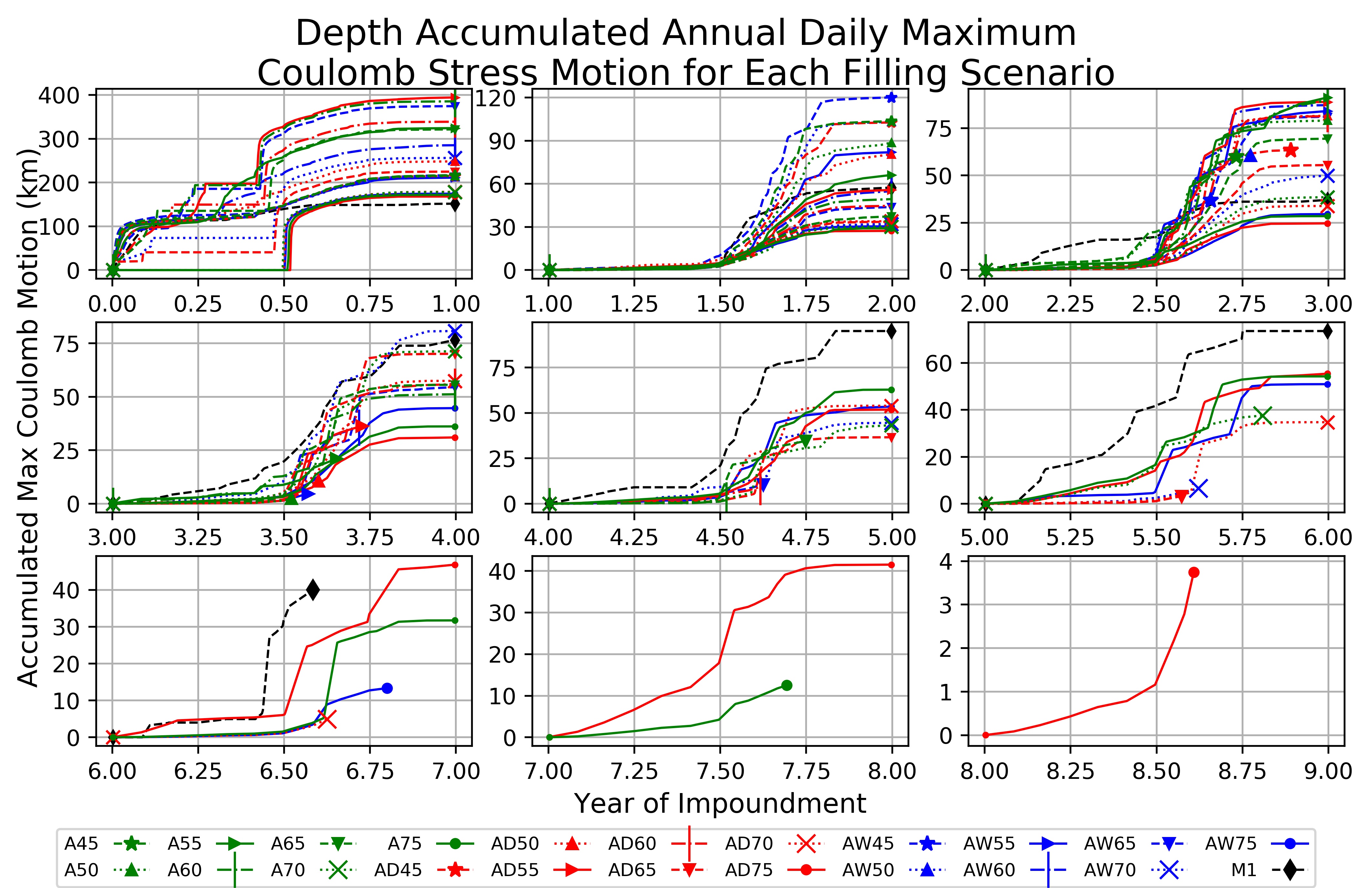
**Online Resource 2.** Daily depth-accumulated movement of the maximum Coulomb stress cell for 22 different filling scenarios. Markers on each of the impoundment scenarios denote the end of the annual period as well as the end of that particular filling scenario. We note that each subplot within the figure is a year's worth of accumulated maximum Coulomb stress distance changes and has its own y-axis limit/scale.


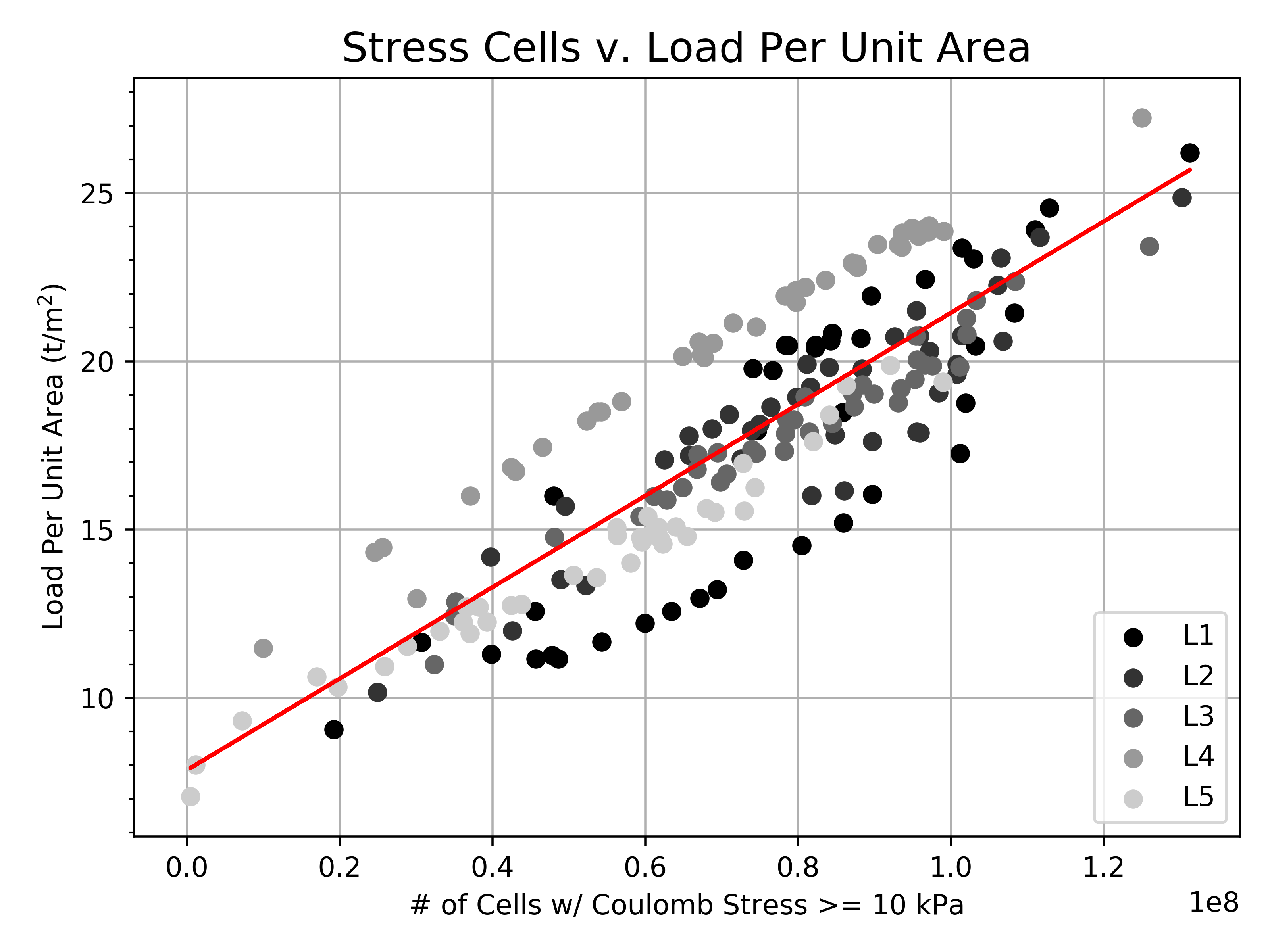


**Online Resource 3.** Depth-accumulated number of cells with a Coulomb stress ≥ 10 kPa against seasonal hydrologic load per unit area for all operational scenarios for each year in the full 39-year operational dataset.


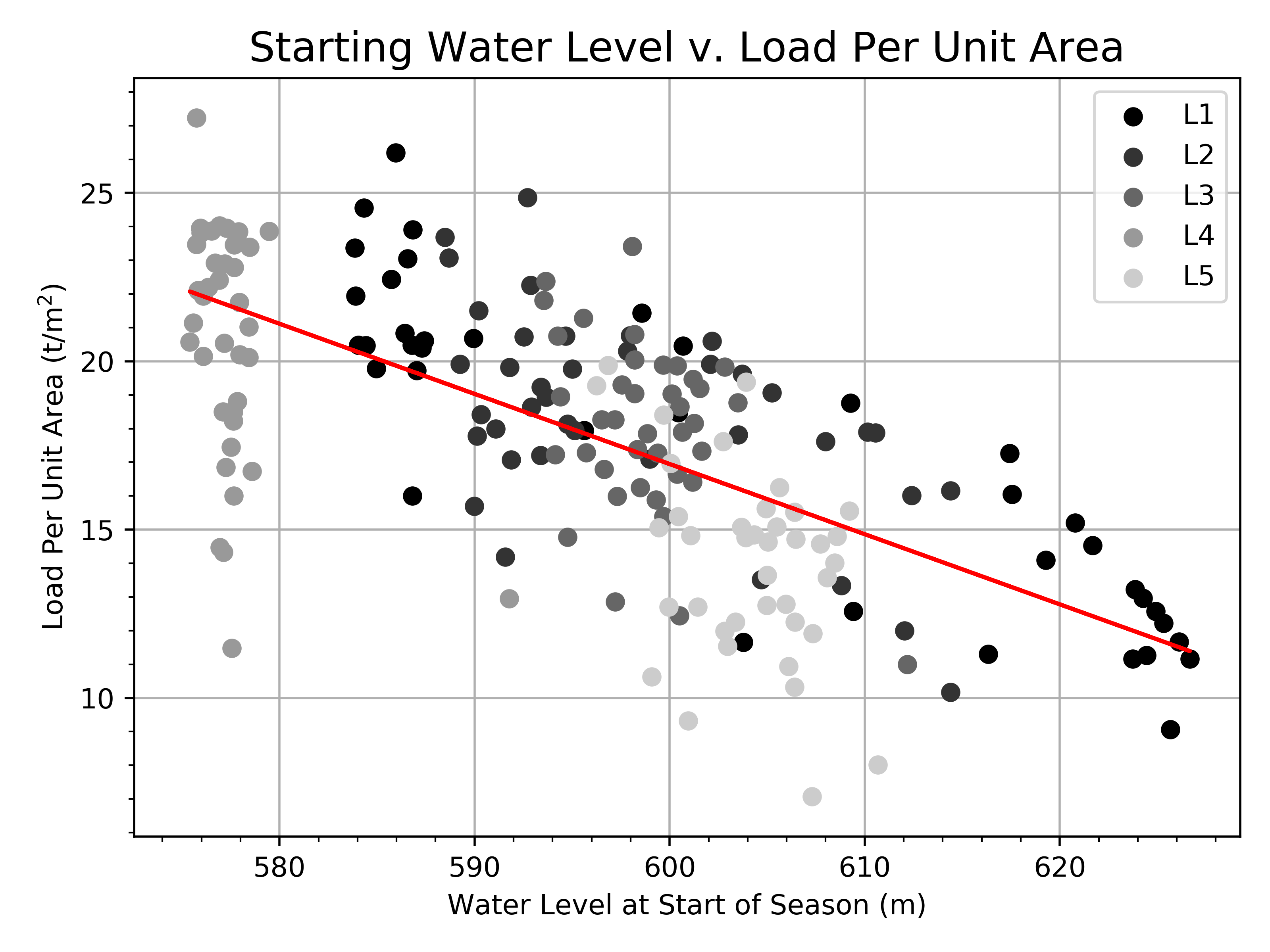


**Online Resource 4.** Water level at seasonal start against seasonal hydrologic load per unit area for all operational scenarios for each year in the full 39-year operational dataset.


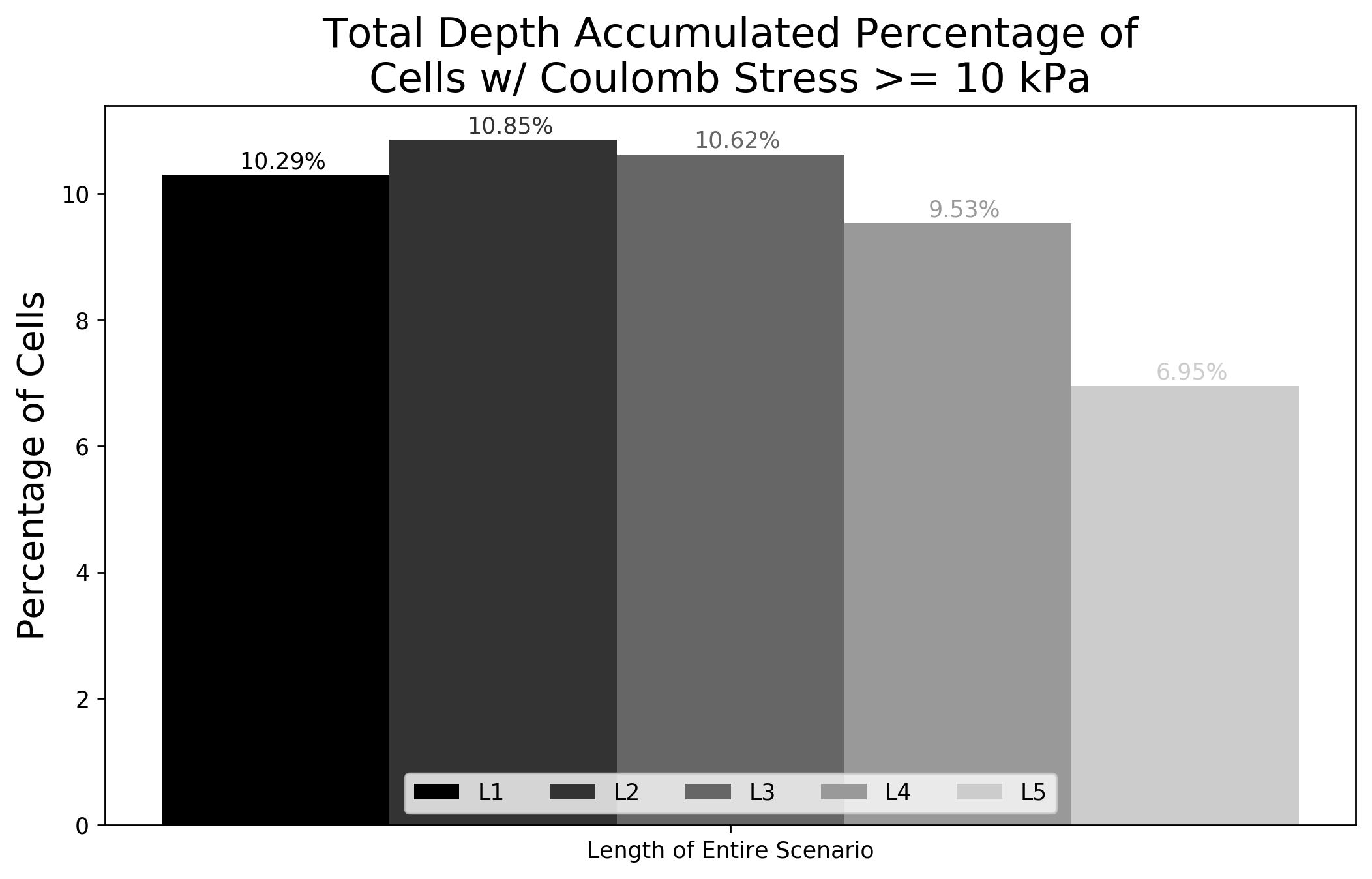


**Online Resource 5.** Total depth-accumulated percentage of cells with a Coulomb stress ≥ 10 kPa for five operational scenarios from the full 39-year operational dataset. The Coulomb stress on optimally oriented fault planes was calculated from hydrologic loads based on the starting water level and the seasonal peak water level for each of the 39 years.


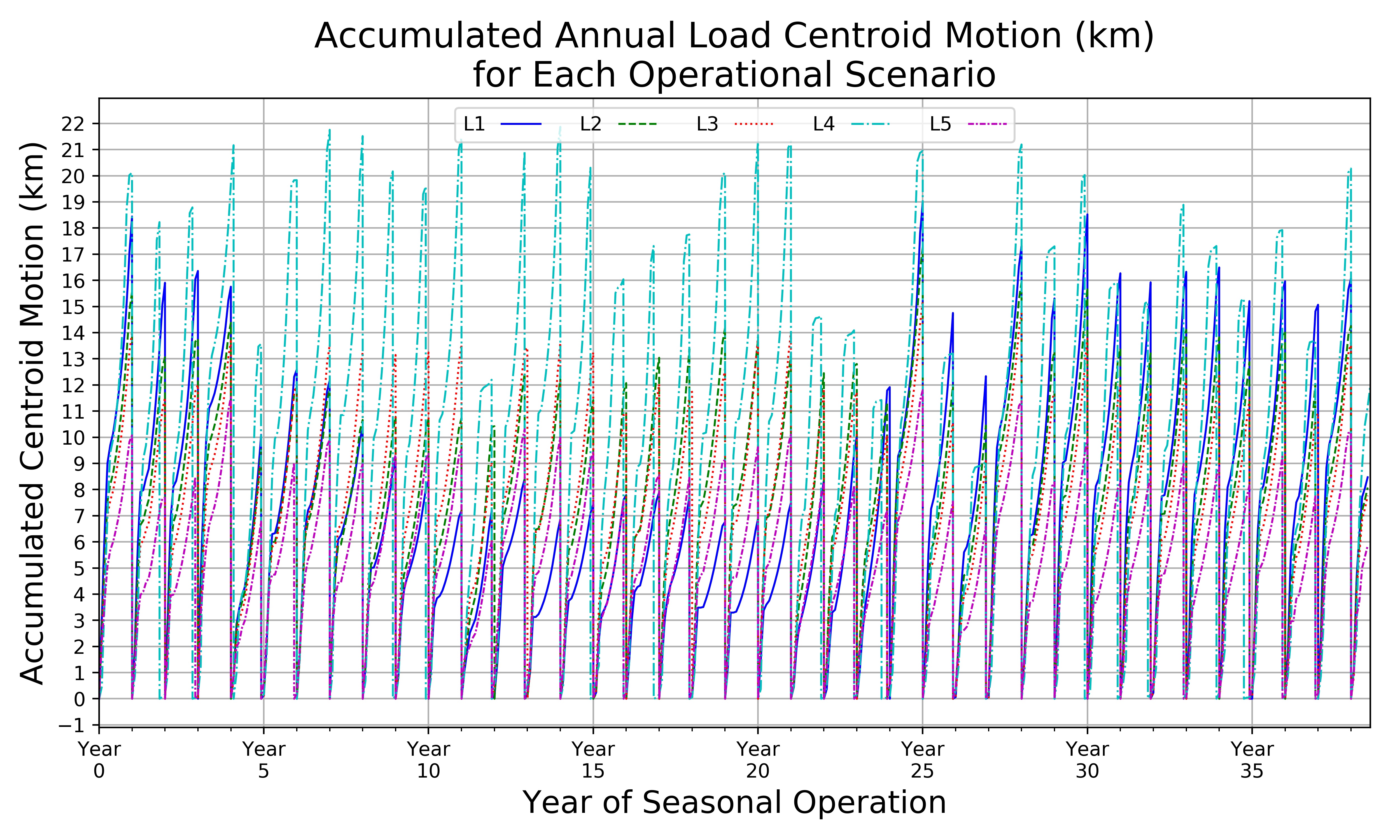


**Online Resource 6.** Seasonal accumulated daily motion of the weighted hydrologic load centroid for the five operational strategies for each year in the full 39-year datasets. These plots track the daily motion of the weighted load centroid for each scenario. The plotted data is the accumulated motion for each seasonal cycle in the full 39-year dataset. Note that the motion at the beginning of each season reverts back to zero, and the subsequent seasonal motion is accumulated from that starting point until the end of the season.

**
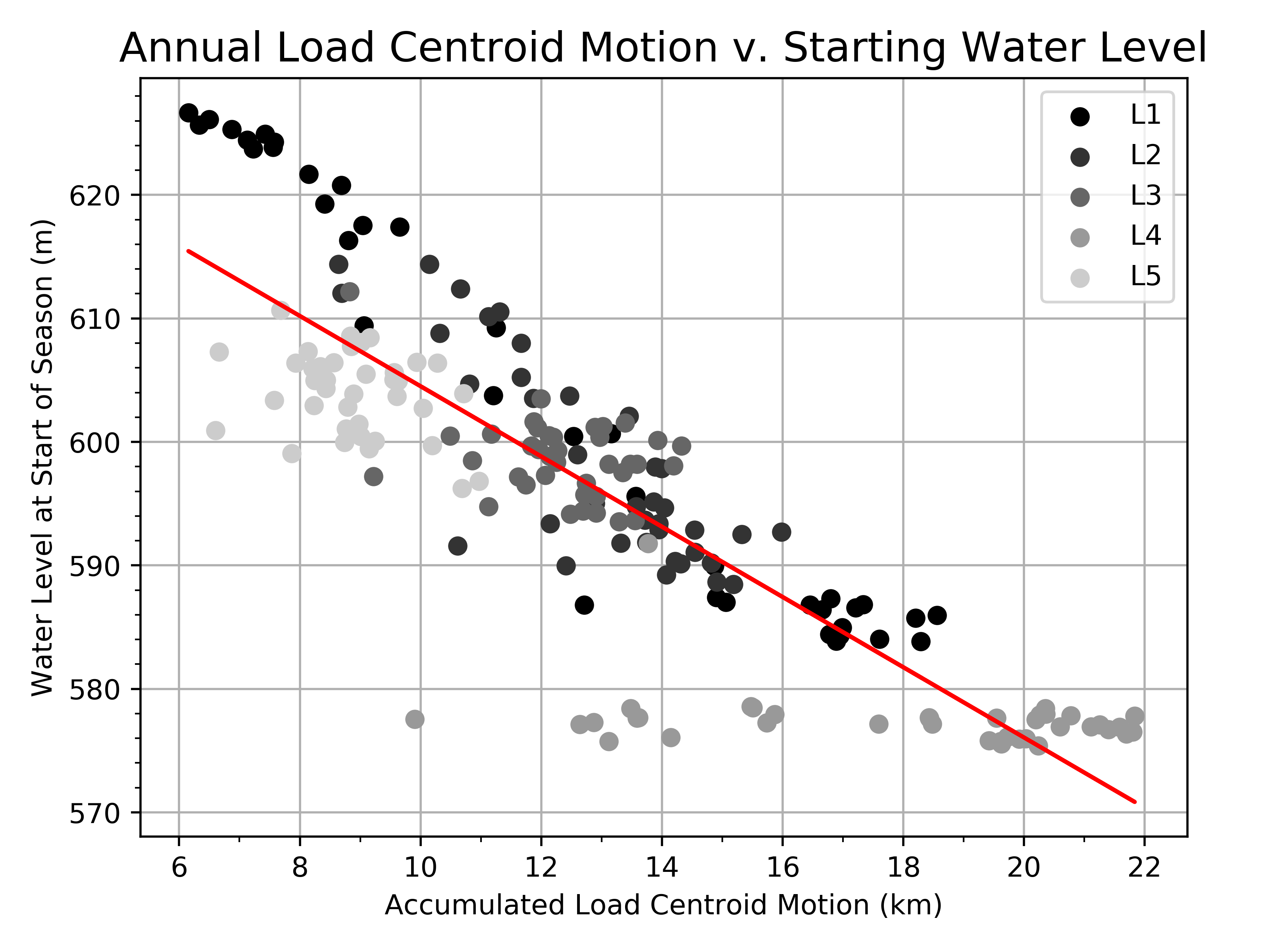
**

**Online Resource 7.** Accumulated annual load centroid motion against water level at seasonal start for all operational scenarios for each year in the full 39-year operational dataset.

Online Resource 8. Animation displaying the Coulomb, normal, and shear stress arrays from the full GERD impoundment for each depth (0 km to 25 m) in our model. The location of the maximum Coulomb stress for each depth is plotted as a white cross and the contour lines denote the location of the 10 kPa Coulomb stress regions.

Online Resource 9. Animation displaying the Coulomb, normal, and shear stress arrays from operational scenario *L1* for the 590 m starting water level at each depth (0 km to 25 m) in our model. The location of the maximum Coulomb stress for each depth is plotted as a white cross and the contour lines denote the location of the 10 kPa Coulomb stress regions.

Online Resource 10. Animation displaying the Coulomb, normal, and shear stress arrays from operational scenario *L2* for the 590 m starting water level at each depth (0 km to 25 m) in our model. The location of the maximum Coulomb stress for each depth is plotted as a white cross and the contour lines denote the location of the 10 kPa Coulomb stress regions.

Online Resource 11. Animation displaying the Coulomb, normal, and shear stress arrays from operational scenario *L3* for the 590 m starting water level at each depth (0 km to 25 m) in our model. The location of the maximum Coulomb stress for each depth is plotted as a white cross and the contour lines denote the location of the 10 kPa Coulomb stress regions.

Online Resource 12. Animation displaying the Coulomb, normal, and shear stress arrays from operational scenario *L4* for the 590 m starting water level at each depth (0 km to 25 m) in our model. The location of the maximum Coulomb stress for each depth is plotted as a white cross and the contour lines denote the location of the 10 kPa Coulomb stress regions.

Online Resource 13. Animation displaying the Coulomb, normal, and shear stress arrays from operational scenario *L5* for the 590 m starting water level at each depth (0 km to 25 m) in our model. The location of the maximum Coulomb stress for each depth is plotted as a white cross and the contour lines denote the location of the 10 kPa Coulomb stress regions.

Online Resource 14. Animation displaying the Coulomb, normal, and shear stress arrays from operational scenario *L1* for the 622 m starting water level at each depth (0 km to 25 m) in our model. The location of the maximum Coulomb stress for each depth is plotted as a white cross and the contour lines denote the location of the 10 kPa Coulomb stress regions.

Online Resource 15. Animation displaying the Coulomb, normal, and shear stress arrays from operational scenario *L2* for the 622 m starting water level at each depth (0 km to 25 m) in our model. The location of the maximum Coulomb stress for each depth is plotted as a white cross and the contour lines denote the location of the 10 kPa Coulomb stress regions.

Online Resource 16. Animation displaying the Coulomb, normal, and shear stress arrays from operational scenario *L3* for the 622 m starting water level at each depth (0 km to 25 m) in our model. The location of the maximum Coulomb stress for each depth is plotted as a white cross and the contour lines denote the location of the 10 kPa Coulomb stress regions.

Online Resource 17. Animation displaying the Coulomb, normal, and shear stress arrays from operational scenario *L4* for the 622 m starting water level at each depth (0 km to 25 m) in our model. The location of the maximum Coulomb stress for each depth is plotted as a white cross and the contour lines denote the location of the 10 kPa Coulomb stress regions.

Online Resource 18. Animation displaying the Coulomb, normal, and shear stress arrays from operational scenario *L5* for the 622 m starting water level at each depth (0 km to 25 m) in our model. The location of the maximum Coulomb stress for each depth is plotted as a white cross and the contour lines denote the location of the 10 kPa Coulomb stress regions.
