# Supplementary figures and images for: Coulomb stress analysis for several filling and operational scenarios at the Grand Ethiopian Renaissance Dam impoundment
Source: Environ Earth Sci. 2021 Mar 28;80(7):286. doi: 10.1007/s12665-021-09591-w (PMC8550559; doi:10.1007/s12665-021-09591-w)

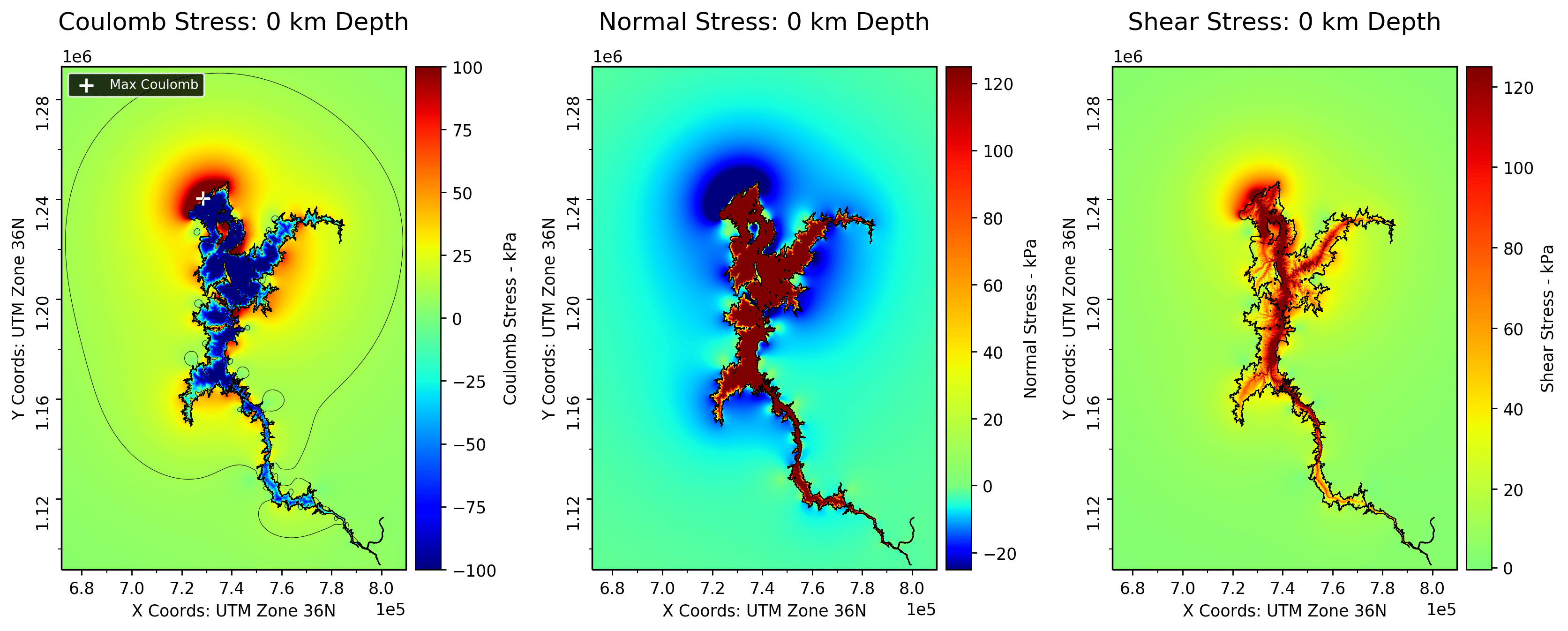

Supplement: Supplementary file 2 — Supplementary file2 (GIF 21818 kb) [file 12665_2021_9591_MOESM2_ESM.gif]

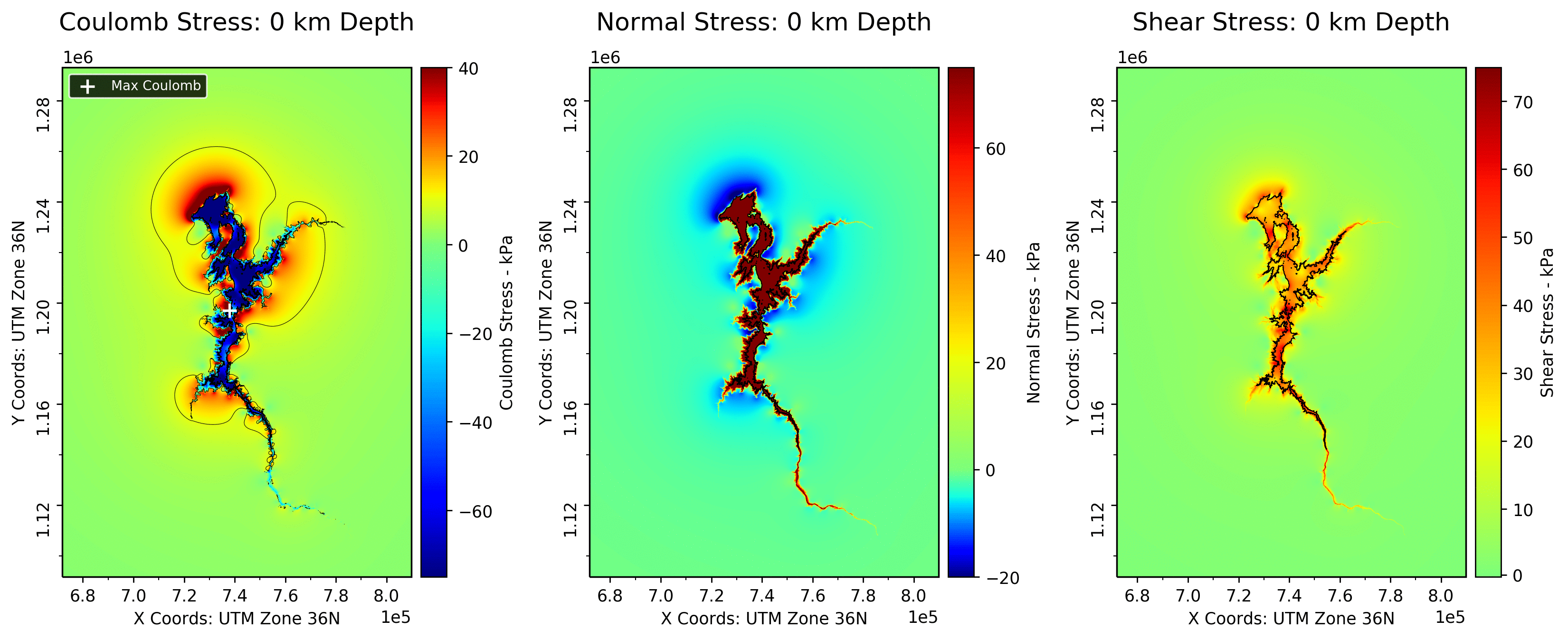

Supplement: Supplementary file 3 — Supplementary file3 (GIF 19512 kb) [file 12665_2021_9591_MOESM3_ESM.gif]

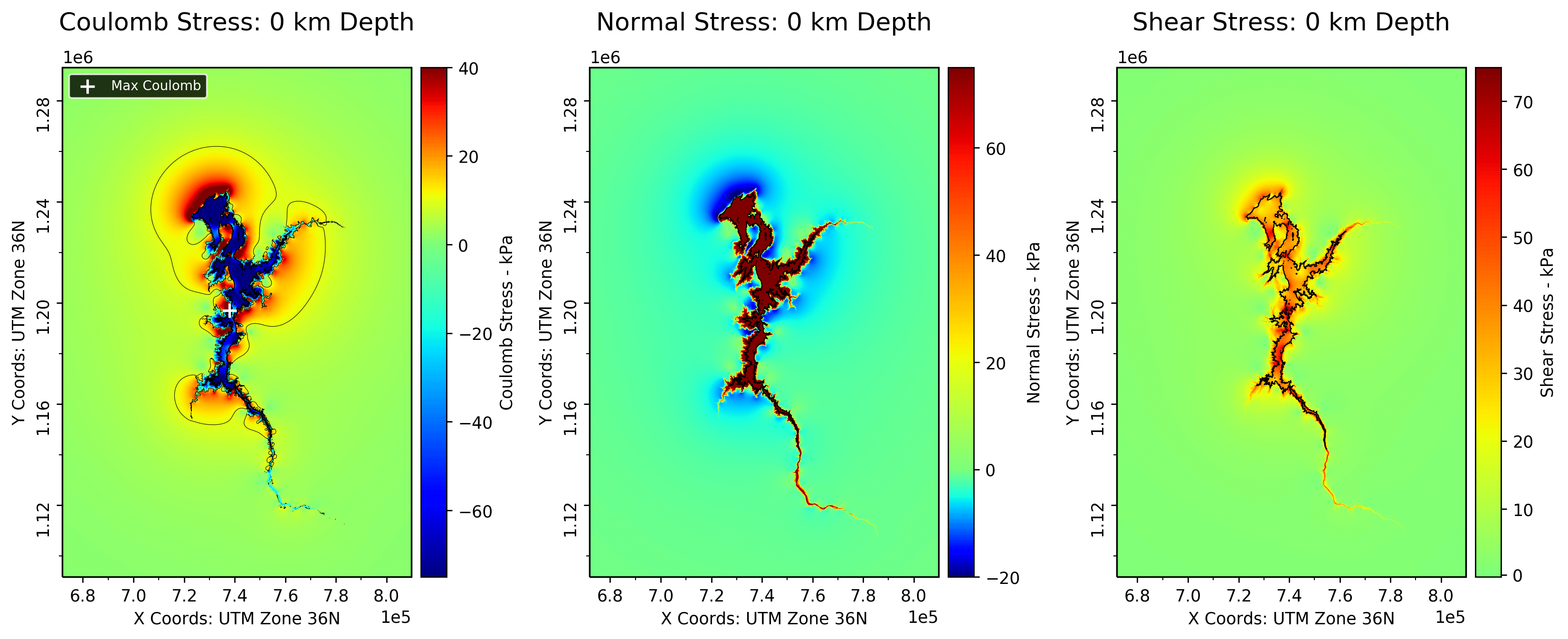

Supplement: Supplementary file 4 — Supplementary file4 (GIF 19545 kb) [file 12665_2021_9591_MOESM4_ESM.gif]

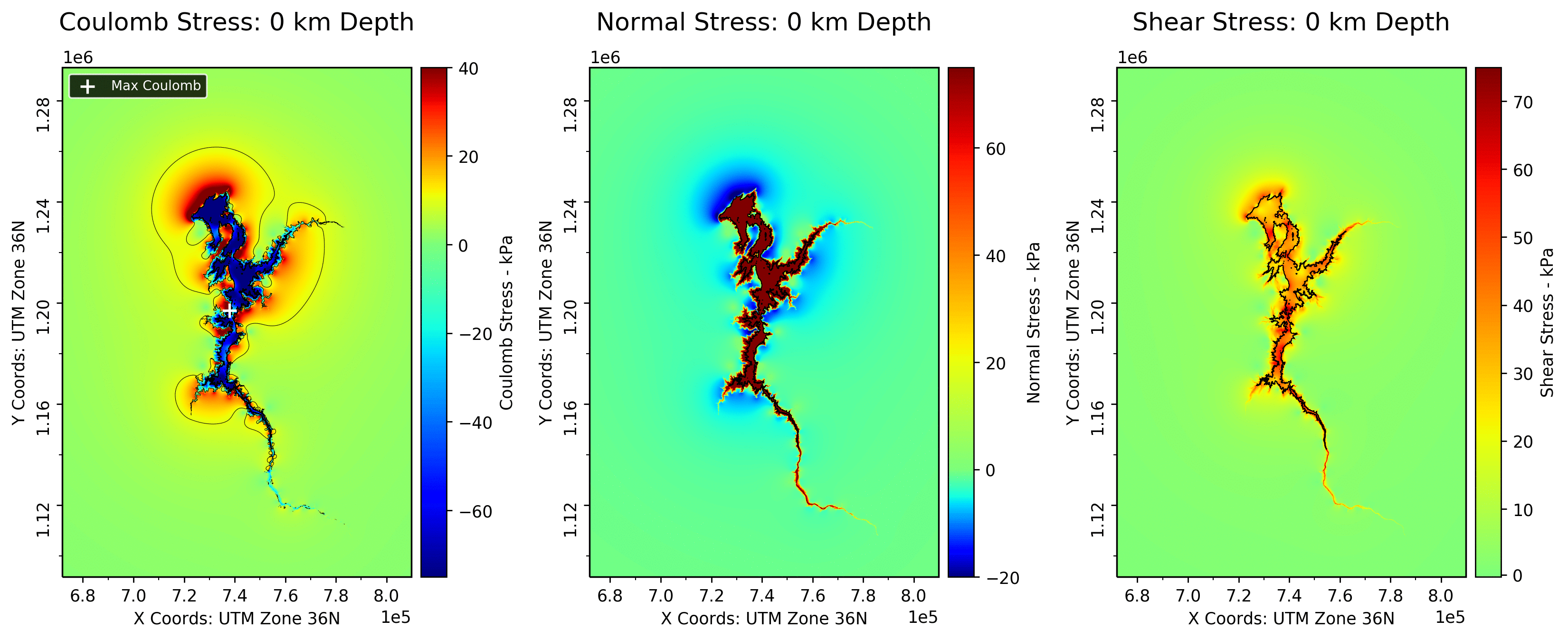

Supplement: Supplementary file 5 — Supplementary file5 (GIF 19419 kb) [file 12665_2021_9591_MOESM5_ESM.gif]

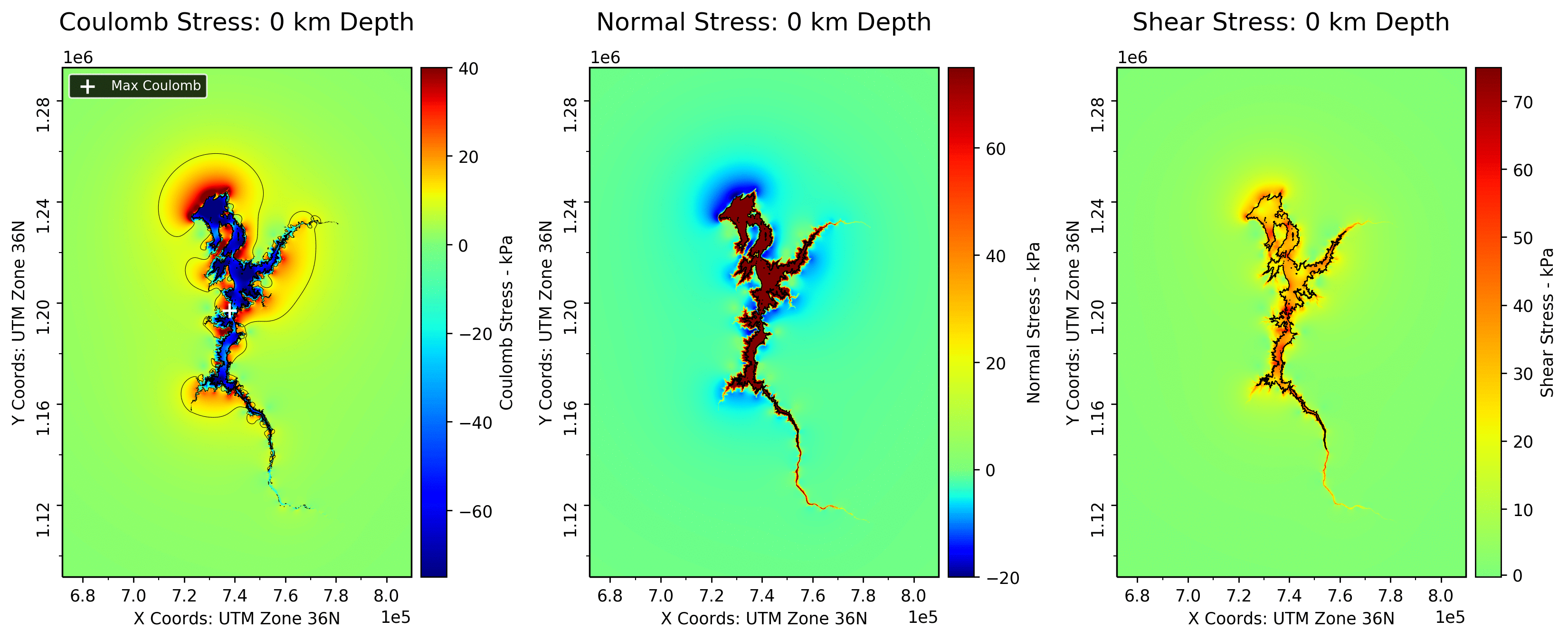

Supplement: Supplementary file 6 — Supplementary file6 (GIF 18659 kb) [file 12665_2021_9591_MOESM6_ESM.gif]

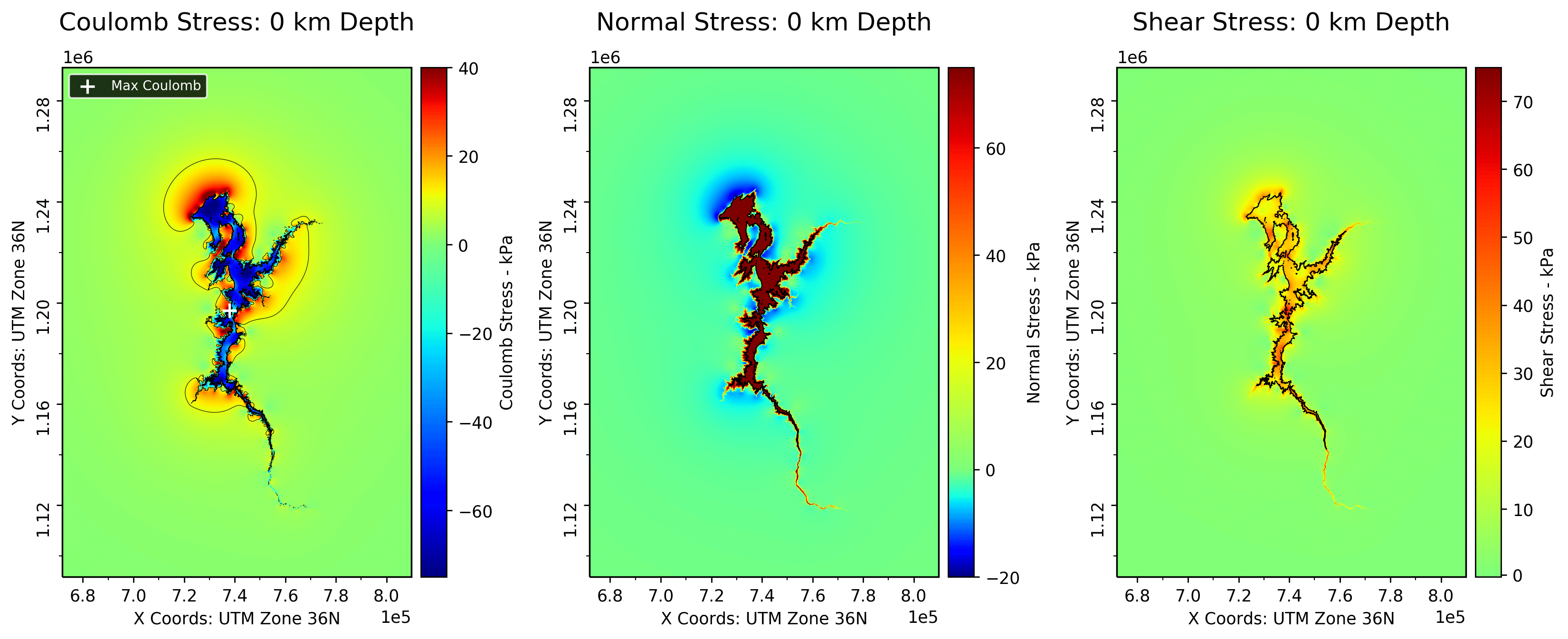

Supplement: Supplementary file 7 — Supplementary file7 (GIF 17870 kb) [file 12665_2021_9591_MOESM7_ESM.gif]

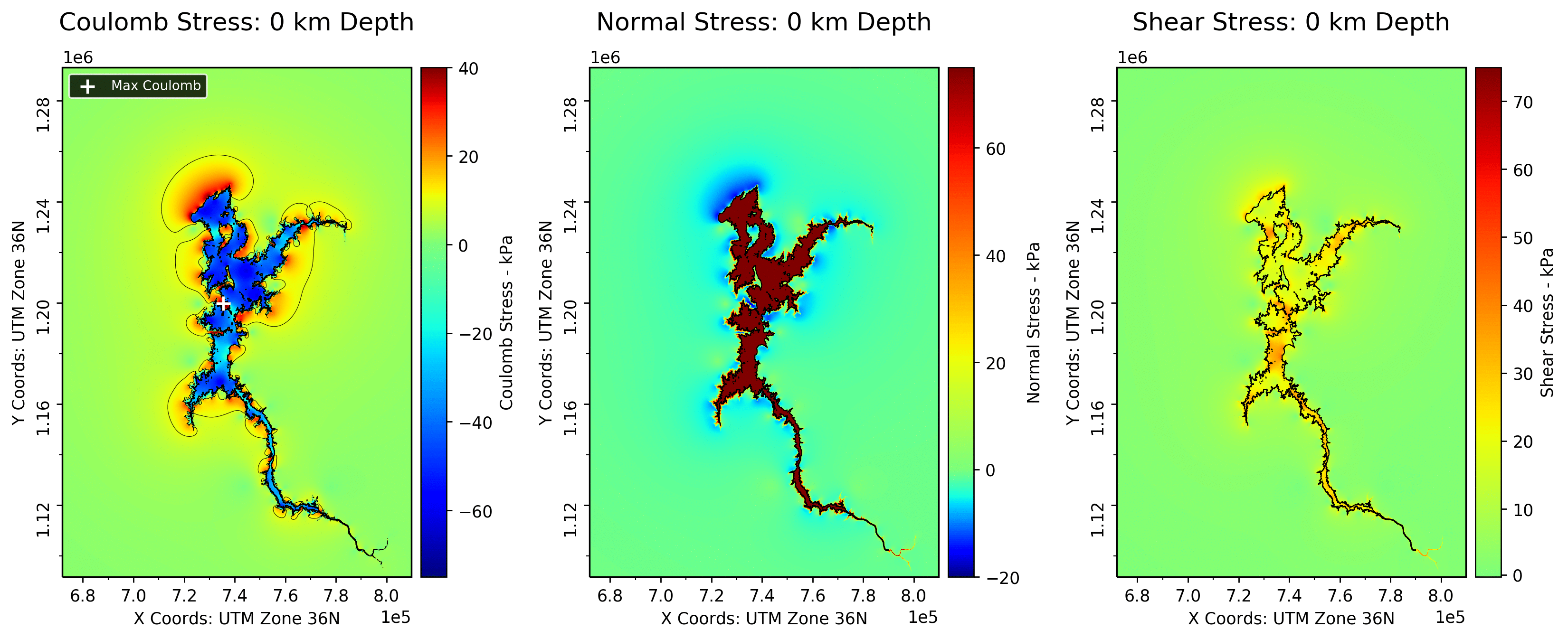

Supplement: Supplementary file 8 — Supplementary file8 (GIF 19748 kb) [file 12665_2021_9591_MOESM8_ESM.gif]

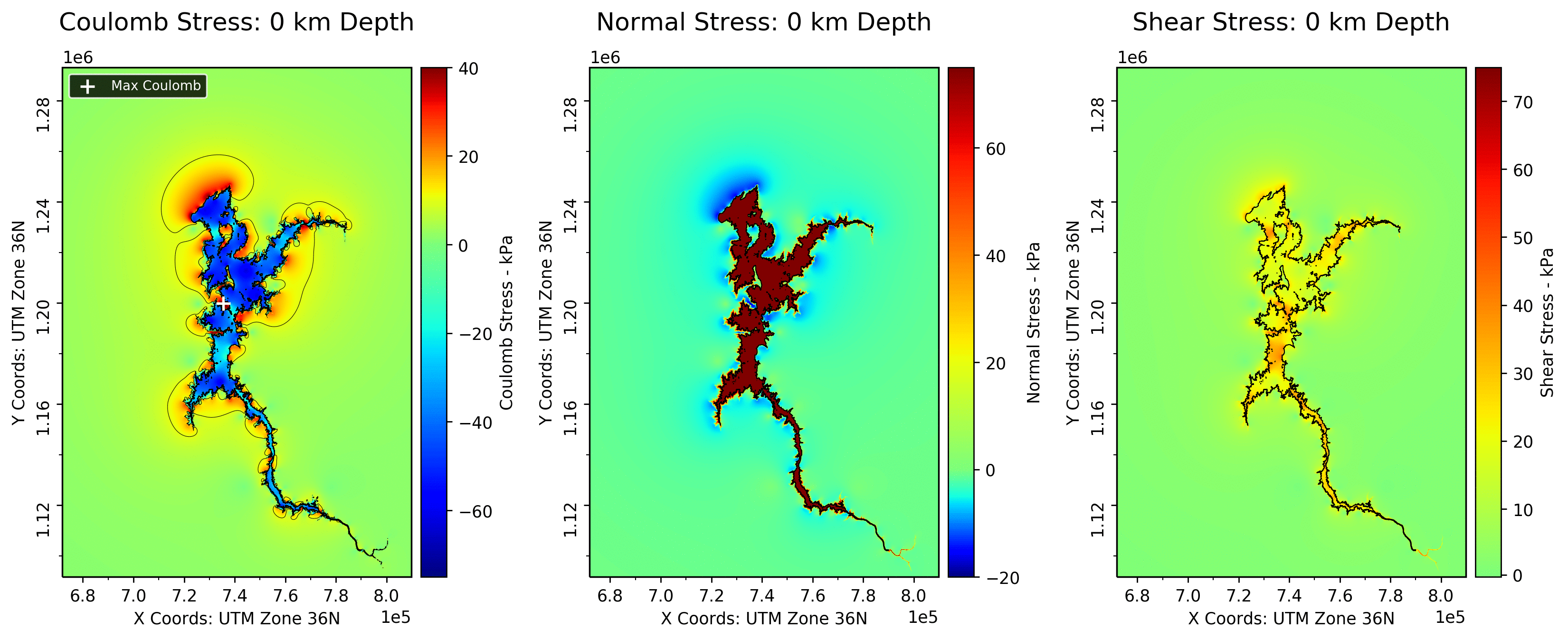

Supplement: Supplementary file 9 — Supplementary file9 (GIF 19786 kb) [file 12665_2021_9591_MOESM9_ESM.gif]

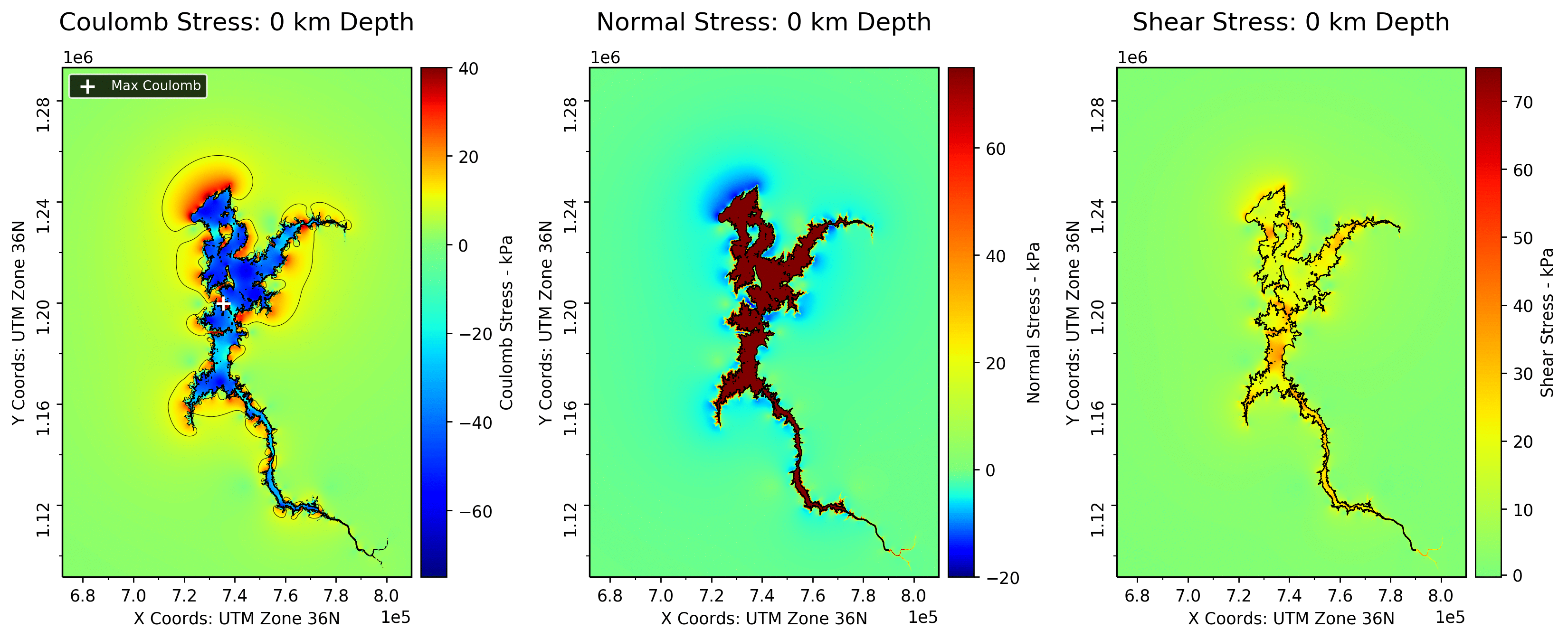

Supplement: Supplementary file 10 — Supplementary file10 (GIF 19740 kb) [file 12665_2021_9591_MOESM10_ESM.gif]

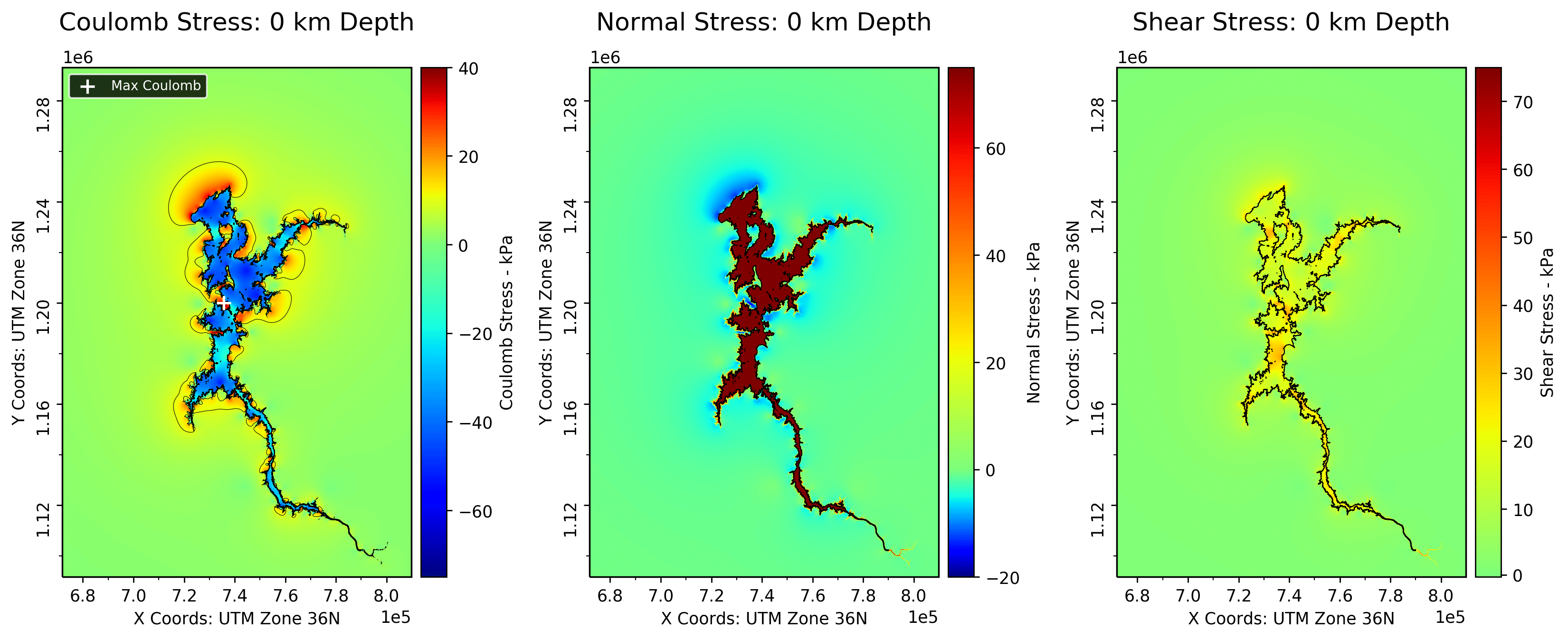

Supplement: Supplementary file 11 — Supplementary file11 (GIF 19124 kb) [file 12665_2021_9591_MOESM11_ESM.gif]

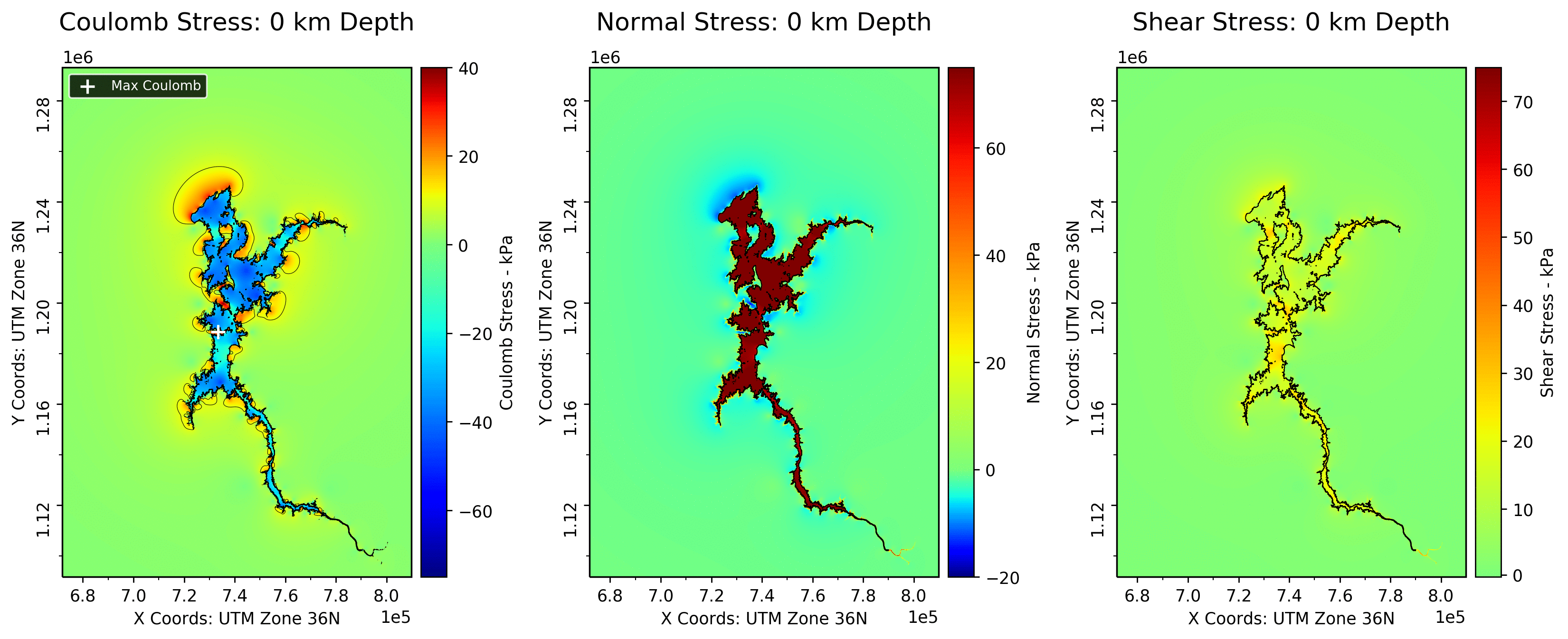

Supplement: Supplementary file 12 — Supplementary file12 (GIF 18531 kb) [file 12665_2021_9591_MOESM12_ESM.gif]
